# Structural Snapshots of *Proteus vulgaris* Tryptophan Indole-Lyase Reveal Insights into the Catalytic Mechanism

**DOI:** 10.1021/acscatal.4c03232

**Published:** 2024-07-18

**Authors:** Robert S. Phillips, S. Meredith Brown, Ravi S. Patel

**Affiliations:** † Department of Chemistry, University of Georgia, Athens, Georgia 30602, United States; ‡ Department of Biochemistry and Molecular Biology, University of Georgia, Athens, Georgia 30602, United States

**Keywords:** pyridoxal-5′-phosphate, reaction mechanism, proton transfer, enzyme dynamics, X-ray crystallography

## Abstract

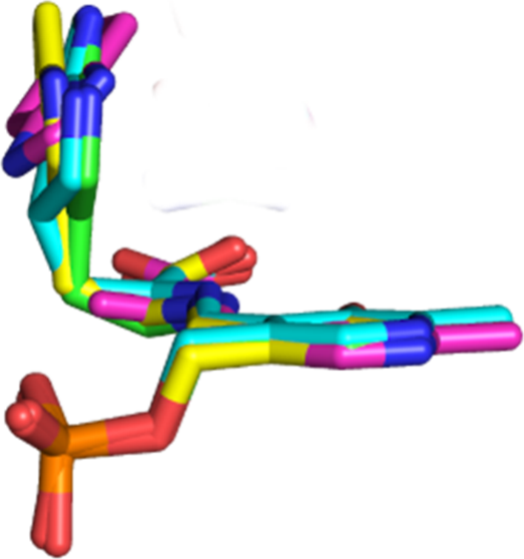

Tryptophan indole lyase (TIL; [E.C. 4.1.99.1]) is a bacterial pyridoxal-5′-phosphate (PLP)-dependent enzyme that catalyzes reversible β-elimination of indole from L-tryptophan. The mechanism of elimination of indole from L-tryptophan starts with the formation of an external aldimine of the substrate and PLP, followed by deprotonation of the α-CH of the substrate, forming a resonance-stabilized quinonoid intermediate. Proton transfer to C3 of the indole ring and carbon–carbon bond cleavage of the quinonoid intermediate provide indole and aminoacrylate bound to PLP, which then releases indole, followed by iminopyruvate. We have now determined the X-ray crystal structures of TIL complexes with (3*S*)-dioxindolyl-l-alanine, an inhibitor, and with substrates L-tryptophan, 7-aza-L-tryptophan, and *S*-ethyl-l-cysteine (SEC) in the presence of benzimidazole (BZI), an isostere of the product indole. These structures show a mixture of *gem*-diamine, external aldimine, quinonoid, and aminoacrylate intermediates, in both open and closed active site conformations. In the closed conformations of L-tryptophan, (3*S*)-dioxindolyl-l-alanine, and 7-aza-L-tryptophan complexes, hydrogen bonds form between Asp-133 with N1 of the ligand heterocyclic ring and NE2 of His-458 in the small domain of TIL. This hydrogen bond also forms in the BZI complex with the aminoacrylate intermediates formed from both L-tryptophan and SEC. The closed quinonoid complex of 7-aza-L-tryptophan shows that the azaindole ring in the closed conformation is bent out of plane of the Cβ–C3 bond by about 40°, putting it in a geometry that leads toward the transition-state geometry. Thus, both conformational dynamics and substrate activation play critical roles in the reaction mechanism of the TIL.

## Introduction

Tryptophan indole lyase (TIL, EC 4.1.99.1) is a pyridoxal-5′-phosphate (PLP)-dependent enzyme that catalyzes the reversible cleavage of L-tryptophan to indole and ammonium pyruvate (eq 1). The enzyme is widely distributed in enterobacteriaceae,^1^ such as *Escherichia coli*,^2^*Klebsiella oxytoca*,^3^
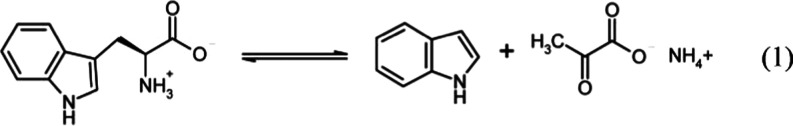
1*Yersinia enterocolitica*,^4^*Haemophilus influenzae*,^5^ and *Proteus vulgaris*,^6^ some of which are healthy gut microflora, but others may result in serious gastrointestinal infections. The enzyme in *E. coli* is induced by exogenous L-tryptophan and is found in a minioperon, which also contains a gene coding for a low-affinity l-Trp transporter.^7^ Indole produced by TIL in these bacteria has been found to have numerous effects on bacterial physiology, including biofilm formation,^8^ antibiotic resistance,^9^ plasmid retention,^10^ and pathogen virulence,^11^ as well as mammalian host health span.^12^ TIL has been recently suggested as a potential drug target for chronic kidney disease.^13^ Furthermore, TIL is involved in the biosynthesis of a cyanobacterial neurotoxin that causes neuropathy in eagles.^14^

**Scheme 1:**
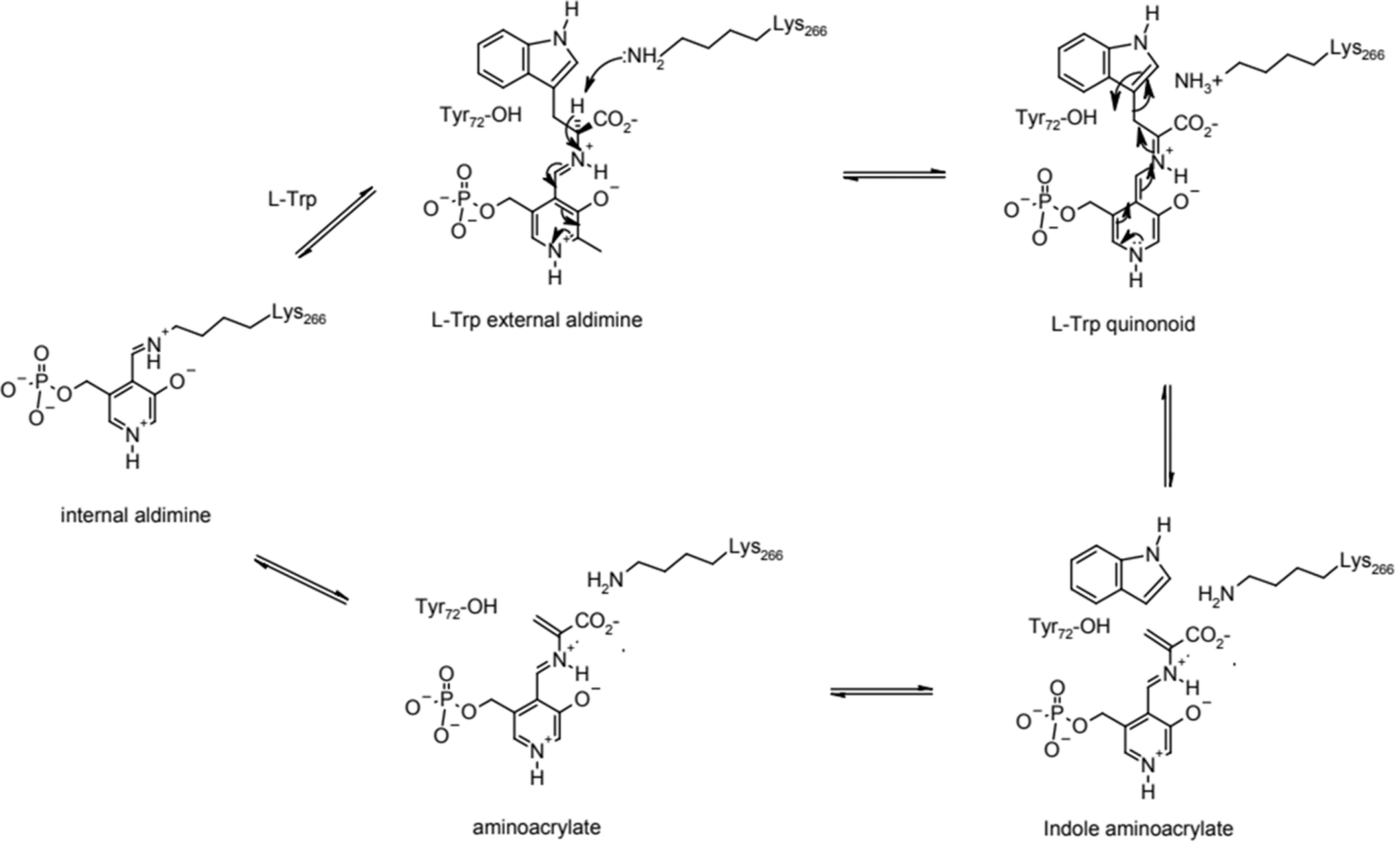
Reaction Mechanism of TIL

**Scheme 2:**
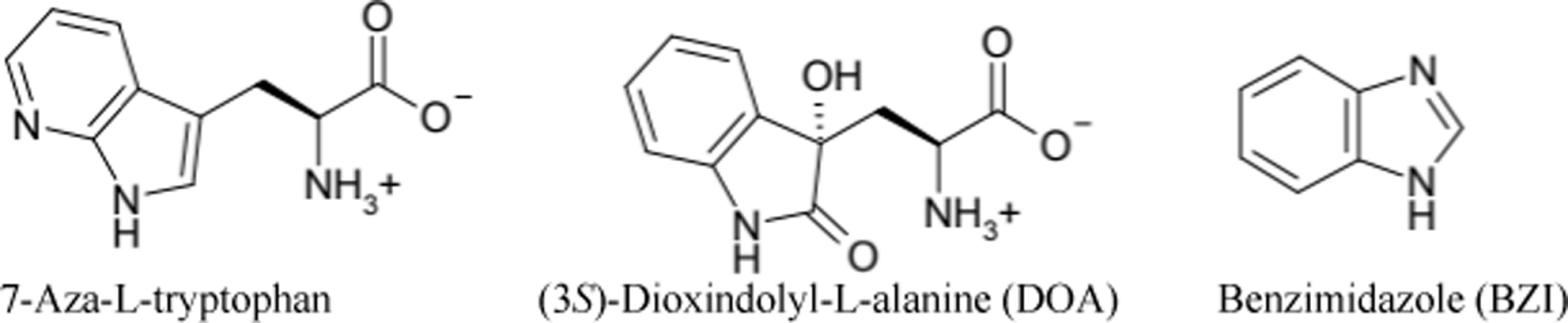
Structures of Tryptophan and Indole Analogues Used in This Study

The reaction mechanism of TIL is of interest since it catalyzes the β-elimination of a carbon–carbon bond with a formally poor leaving group, indole. Furthermore, the reaction in eq 1 is reversible, and TIL can synthesize l-Trp from indole and ammonium pyruvate.^15^ Hence, TIL has also been used for the synthesis of halogenated tryptophans from halogenated indoles.^16^ Our previous stopped-flow kinetic studies have shown that an external aldimine of l-Trp with PLP forms rapidly (Scheme 1). A quinonoid complex is then formed by Cα-deprotonation, which subsequently undergoes elimination of indole, concomitant with proton transfer from Tyr-72 to C3 of the indole ring, to form a PLP-aminoacrylate complex.^17^ Inhibition of TIL by oxindolyl-l-alanine and 2,3-dihydro-L-tryptophan had suggested that an indolenine intermediate is formed in the mechanism.^18,19^ A crystal structure of *P. vulgaris* TIL with oxindolyl-l-alanine bound has been obtained previously, showing that the complex is a quinonoid structure and that the active site is in a closed conformation.^20^ Presteady-state primary and secondary isotope effects on the elimination of indole from l-Trp suggested a concerted step of Cβ–C3 carbon–carbon bond cleavage and proton transfer to C3 of the indole, with an indolenine-like transition state, rather than an intermediate.^21^ However, the crystal structures of most of the proposed reaction intermediates have not been obtained previously. We have now obtained the crystal structures of *P. vulgaris* TIL complexed with 7-aza-l-Trp (Scheme 2), a slow substrate, (3*S*)-dioxindolyl-l-alanine ((3*S*)-DOA), a potent competitive inhibitor, and good substrates, l-Trp and *S*-ethyl-l-cysteine, in the presence of benzimidazole (BZI), an inhibitor that is an isostere of indole. These structures exhibit equilibrating mixtures of *gem*-diamine, external aldimine, quinonoid, and aminoacrylate complexes of the ligands, in both open and closed active site conformations, illustrating the structural dynamics of TIL during the catalytic cycle. In addition, the quinonoid complex of TIL with 7-aza-L-tryptophan shows clear evidence for bending of the azaindole ring of the substrate, demonstrating the activation of the substrate for catalysis.

## Results

### Structure of the TIL Complex with 7-Aza-L-tryptophan

**Figure 1:**
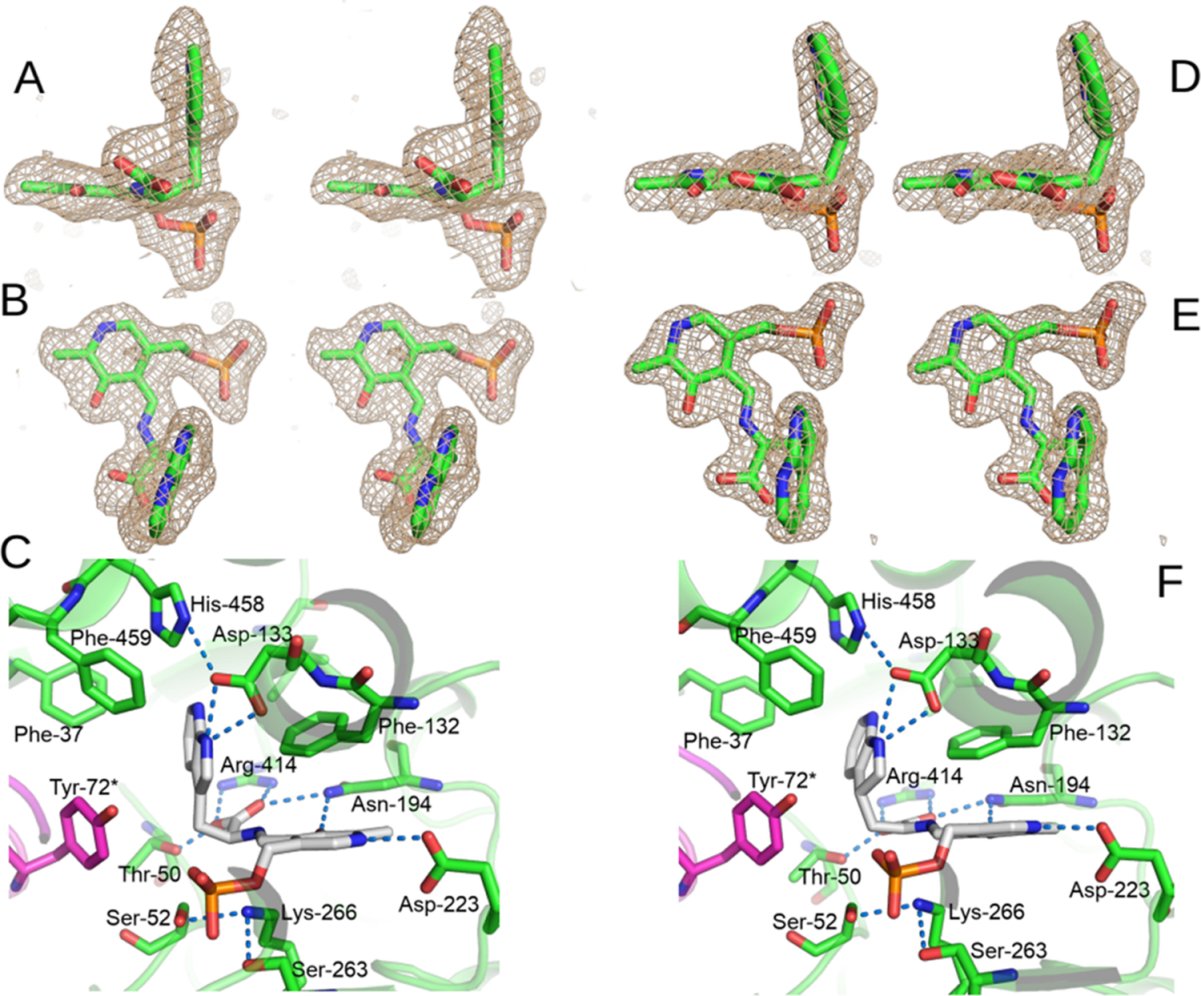
(A) Stereo side view of the sim omit mFo-DFc map at 3σ of the PLP-7-aza-l-Trp external aldimine complex in chain A. (B) Stereo top view of the sim omit mFo-DFc map at 3σ of the PLP-7-aza-l-Trp external aldimine complex in chain A. (C) View of the environment of the PLP-7-aza-l-Trp external aldimine complex in chain A, showing potential hydrogen bonds with blue dashes. The hydrogen bonds of the phosphate have been omitted for clarity. (D) Stereo side view of the sim omit mFo-DFc map at 3σ of the PLP-7-aza-l-Trp quinonoid complex in chain D. (E) Stereo top view of the sim omit mFo-DFc map at 3σ of the PLP-7-aza-l-Trp quinonoid complex in chain D. (F) View of the environment of the PLP-7-aza-l-Trp quinonoid complex in chain D, showing potential hydrogen bonds with blue dashes. The hydrogen bonds of the phosphate have been omitted for clarity.

7-Aza-l-Trp is a very slow substrate for TIL, with *k*_cat_ of 0.04 s^–1^, about 1% that of l-Trp.^22,23^ In solution, 7-aza-l-Trp forms an absorbance peak with TIL with λ_max_ at 498 nm, with a rate constant of 140 s^–1^, showing that a quinonoid complex is formed rapidly,^22,23^ despite the slow steady-state reaction. This encouraged us to determine the crystal structure of TIL soaked with 7-aza-l-Trp, in order to obtain the structures of reaction intermediates. Hence, crystals of TIL soaked in a cryosolvent with 50 mM 7-aza-l-Trp turned orange, demonstrating that a quinonoid complex is also formed in the crystals. The crystal structure of the complex was determined to a resolution of 1.74 Å, with *R*_work_/*R*_free_ of 18.5%/22.8%, in space group *P*2_1_2_1_2 (**8V6P**, Table 1). The functional assembly of TIL, and the asymmetric unit, is a homotetramer. The active site is formed in a dimer at the interface of two monomers. The enzyme in the absence of ligands is in an open conformation. In the closed conformation, the α-helix1 from residues 20–38, the α-helix4, β-turn, and β-sheet from 108 to 129, the β-turn and α-helix13 from 361 to 389, an extended β-turn from 389 to 414, and residues 439–467, which form a complicated α-helix15–β-turn–β-sheet–α-helix16–β-sheet structure in the small domain, rotate toward the large domain, moving as much as 8 Å.^20^ Three of the subunits (A, B, and D) of this structure are in a fully closed conformation, while chain C has a disordered small domain (Figure S1). Hence, chain C was built and refined as a mixture of open and closed conformations for the small domain and gave occupancies of ∼60% open and ∼40% closed (Figure S2). Clear density for the bound ligand was observed in all four active sites, and there is no remaining electron density between NZ (the ε-nitrogen) of Lys-266 and the PLP in any of the sites, showing that the transfer of PLP from the internal aldimine to the ligand is complete. The structures of the bound ligands were assigned as either external aldimine or quinonoid complexes by examining the carboxylate of the bound amino acid. If the carboxylate is approximately planar with the PLP ring, then the complex was assigned as a quinonoid (Figure 1D).

**Table 1:** Data Collection and Refinement Statistics^a^

	TIL-7-Aza-l-Trp (**8V6P**)	TIL-(3*S*)-DOA (**8V9P**)	TIL-l-Trp-BZI (**9BLV**)	TIL-S-Et-l-Cys-BZI (**9BNJ**)
wavelength	1.0000 A	0.99999 A	1.0000 A	0.97856 A
ellipsoidal resolution range	52.29–1.74 (1.92–1.74)	61.87–1.845 (2.123–1.845)	56.76–1.776 (1.80–1.776)	
ellipsoidal resolution limits	a* = 1.93, b* = 1.74, c* = 2.19	a* = 2.93, b* = 2.13, c* = 1.84	a* = 1.88, b* = 2.96, c* = 1.78	
spherical resolution range	52.29–1.89 (1.92–1.89)	61.87–2.169 (2.206–2.169)	56.76–1.83 (1.86–1.83)	82.04–1.512 (1.53–1.51)
space group	*P*2_1_2_1_2	*P*_1_2_1_1	*P*2_1_2_1_2_1_	*P*2_1_2_1_2_1_
unit cell	151.635, 211.34	67.3419, 112.717	113.523, 109.962	113.945, 118.225
	60.956	119.518	144.982	152.47
	90 90 90	90 97.91 90	90 90 90	90 90 90
total reflections	9,53,925 (43,952)	5,00,834 (23,198)	5,46,851 (27,551)	34,05,737 (1,72,746)
unique reflections	1,37,266 (6863)	76,925 (3846)	94,968 (4748)	3,19,496 (15,832)
multiplicity	6.9 (6.4)	6.5 (6.0)	5.8 (5.8)	10.7 (10.9)
completeness, spherical (%)	68.0 (13.0)	50.7 (7.4)	54.4 (10.4)	99.41 (93.53)
completeness, ellipsoidal (%)	93.6 (66.1)	91.3 (57.9)	94.4 (64.1)	
mean I/sigma(I)	5.6 (2.0)	5.1 (1.7)	5.4 (1.6)	7.2 (0.54)
Wilson *B*-factor	21.22	12.03	19.86	20.44
CC(1/2)	0.992 (0.59)	0.980 (0.47)	0.994 (0.666)	0.998 (0.327)
CC*	0.999 (0.618)	0.886 (0.626)	0.998 (0.715)	0.999 (0.558)
reflections used in the refinement	1,37,266 (6863)	76,841 (172)	94,874 (48)	3,17,641 (9853)
reflections used for *R*-free	6814 (59)	3856 (8)	4846 (3)	15,879 (510)
*R*-work	0.1851 (0.3325)	0.1990 (0.365)	0.1975 (0.3153)	0.1579 (0.3082)
*R*-free	0.2279 (0.2912)	0.2466 (0.5115)	0.2473 (0.6909)	0.1798 (0.3236)
number of nonhydrogen atoms	16,246	16,299	15,826	18,938
macromolecules	14,933	15,068	14,758	16,998
ligands	124	116	132	165
solvent	1189	1115	936	1775
protein residues	1853	1868	1839	1854
RMS (bonds)	0.070	0.002	0.005	0.004
RMS (angles)	2.00	0.52	0.70	0.73
Ramachandran favored (%)	95.77	96.55	96.22	96.20
Ramachandran allowed (%)	4.07	3.45	3.78	3.52
Ramachandran outliers (%)	0.16	0.00	0.00	0.27
rotamer outliers (%)	1.21	0.96	0.91	1.01
clashscore	4.80	4.67	6.06	2.12
average *B*-factor	26.81	19.27	33.57	33.41
macromolecules	26.59	19.15	33.76	32.86
ligands	22.22	26.16	30.07	30.69
solvent	29.99	20.79	31.05	38.94
number of TLS groups	19	25	19	20

a Statistics for the highest-resolution shell are shown in parentheses.

If the carboxylate projects above the ring plane, then the complex was assigned as an external aldimine (Figure 1A). On that basis, chains A and C have the 7-aza-l-Trp bound as an external aldimine, while chains B and D have it bound as a quinonoid complex.

The external aldimine of 7-aza-l-Trp in chain A is shown in Figure 1A–C. The electron density around the Cα and the ligand carboxylate is clearly nonplanar with that of the PLP (Figure 1A,B). The carboxylate accepts hydrogen bonds from OG of Thr-50 (2.4 Å), ND2 of Asn-194 (3.2 Å), and NH1 and NH2 of Arg-414 (2.8 and 2.8 Å, respectively) (Figure 1C). ND2 of Asn-194 also donates a hydrogen bond to 3′-O of the PLP (2.8 Å). Asp-223 accepts a hydrogen bond from protonated PLP N1 (2.8 Å). NZ of Lys-266 forms hydrogen bonds with OG of Ser-52 (3.0 Å) and Ser-263 (2.8 Å) and is 3.3 Å from the O20 of the PLP phosphate and 3.6 Å from the Cα of the 7-aza-l-Trp. The azaindole ring is in plane with the C3–Cβ bond, the CZ of Phe-459 is 3.1 Å from N1 of the azaindole, and the OH of Tyr-72* (coming from chain B of the catalytic dimer) is 3.9 Å from C-3. Asp-133 is rotated into the active site, forming hydrogen bonds of OD1 and OD2 with N1 of the azaindole (2.8 and 2.6 Å, respectively) and OD2 with NE2 of His-458 (3.1 Å). Phe-132, which sits atop the pyridine ring of PLP in the internal aldimine, is in a rotated-out conformation, where it sits behind the azaindole ring at 3.6 Å, forming a nearly perpendicular π–π interaction (Figure 1C).

**Figure 2:**
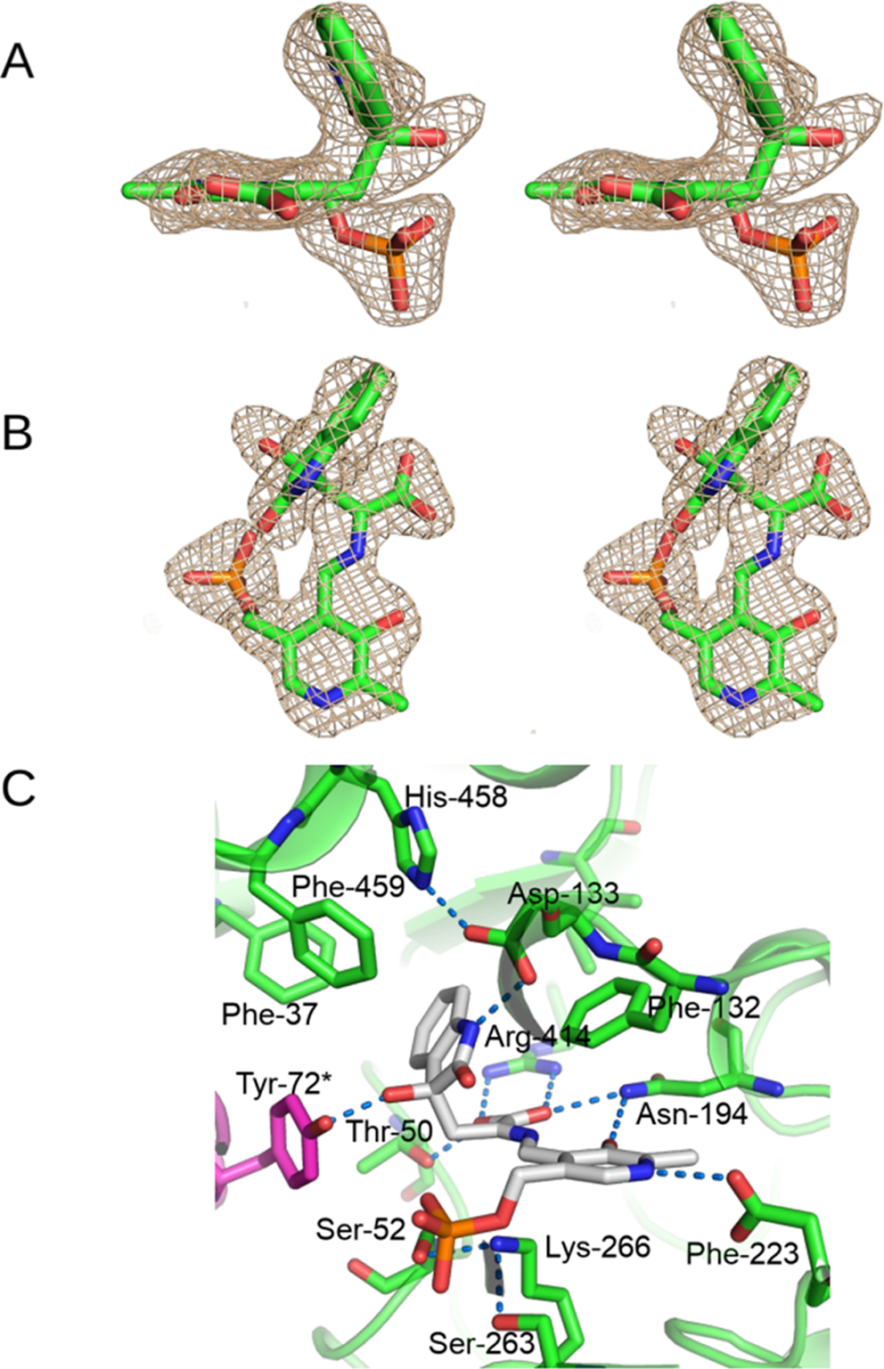
(A) Stereo side view of the sim omit mFo-DFc map at 4σ of the PLP-(3*S*)-dioxindolyl-l-alanine quinonoid complex in chain B. (B) Stereo top view of the sim omit mFo-DFc map at 4σ of the PLP-(3*S*)-dixindolyl-l-alanine quinonoid complex in chain B. (C) Stereo view of the environment of the PLP-(3*S*)-dixindolyl-l-alanine quinonoid complex in chain B, showing potential hydrogen bonds with blue dashes. The hydrogen bonds of the phosphate are omitted for clarity.

The quinonoid complex of 7-aza-l-Trp in chain D is shown in Figure 1D–F. As with the external aldimine, the carboxylate of the quinonoid complex accepts hydrogen bonds from OG of Thr-50 (2.4 Å), ND2 of Asn-194 (2.9 Å), and NH1 and NH2 of Arg-414 (2.7 and 2.6 Å, respectively; Figure 2C). The 3′-O of the PLP also accepts a hydrogen bond from ND2 of Asn-194 (2.9 Å), and N1 of the PLP donates a hydrogen bond to OD1 of Asp-223 (2.7 Å). NZ of Lys-266 forms hydrogen bonds with OG of Ser-52 (2.9 Å) and Ser-263 (3.0 Å) and is 3.5 Å from O20 of the PLP phosphate, and 3.7 Å from the Cα of the 7-aza-l-Trp. Different from the external aldimine, the azaindole ring is clearly nonplanar, bent about 40° from the plane of the Cβ-Cγ bond (Figure 1D,E), and the CZ of Phe-459 is 3.1 Å from N1 of the azaindole, forming a nearly perpendicular π–π interaction. The OH of Tyr-72*, from chain C, has moved closer, and is 3.3 Å from C3 of the azaindole ring. We attempted to refine the quinonoid complexes of 7-aza-L-tryptophan with planar azaindole ring restraints, but it resulted in severe bond angle distortion in the C3–Cβ-Cα angle, and in the PLP ring. These distortions were seen in both sites containing the quinonoid complex, but not for those with the external aldimine. In contrast, refinement of the azaindole ring of the quinonoid complex with nonplanar ring restraints results in no significant angle distortion of these bonds. Asp-133 is in the rotated-in conformation, forming hydrogen bonds of OD1 and OD2 with N1 of the azaindole (3.0 and 3.0 Å) and NE2 of His-458 (3.1 Å), and Phe-132 remains in the rotated-out conformation, where it sits behind the azaindole ring at 3.6 Å, forming a π–π interaction (Figure 1F). In the other quinonoid complex, in chain B, Asp-133 is in a different conformation, with OD1 accepting a hydrogen bond from N1 of the azaindole (3.2 Å) and OD2 accepting a hydrogen bond from N7 of the azaindole (2.9 Å), while OD2 is 3.1 Å from NE2 of His-458.

### Structure of the TIL Complex with (3*S*)-Dioxindolyl-l-alanine

Crystals of TIL were soaked with (3*S*)-DOA, a competitive inhibitor with *K*_i_ = 10 ± 3 μM, and turned dark orange. This structure was solved in space group *P*2_1_ to a resolution of 1.85 Å, with a *R*_work_/*R*_free_ of 19.9%/24.7% (**8V9P**, Table 1). In this structure, all four chains are in a fully closed conformation (Figure S3), similar to what was seen before with oxindolyl-l-alanine.^20^ The complexes of (3*S*)-DOA with PLP show clear density for the bound ligand without any residual density of PLP linked with Lys-266. All of these structures were identified as quinonoid complexes, using the method described above, and all of the sites have similar structures (Figure 2A,B). Similar to 7-aza-l-Trp complexes, the carboxylate of the ligand in chain B forms hydrogen bonds with NH1 and NH2 of Arg-414 (2.7 and 2.7 Å, respectively), OG of Thr-50 (2.5 Å), and ND2 of Asn-194 (2.8 Å) (Figure 2C). ND2 of Asn-194 is also hydrogen-bonded (2.9 Å) to the 3′-O of the PLP, and N-1 of the PLP is hydrogen-bonded to the OD2 of Asp-223 (2.9 Å). NZ of Lys-266 donates hydrogen bonds to OG of Ser-52 (2.8 Å) and OG of Ser-263 (2.9 Å) and is 3.6 Å from the PLP phosphate, O23, and 3.7 Å from Cα. The N-1 of (3*S*)-DOA donates a hydrogen bond to OD1 of Asp-133 (2.7 Å), and OD2 accepts a hydrogen bond from NE2 of His-458 (2.8 Å), while the 3-OH of the dioxindole accepts a hydrogen bond from NH2 of Arg 101 (3.0 Å) and forms a hydrogen bond with the OH of Tyr-72* (2.2 Å). This very short O–H–O distance (2.2–2.4 Å) is seen in all four chains, showing that a short hydrogen bond is formed. It is possible that the OH of Tyr-72* is ionized to the phenolate to better accommodate this short hydrogen bond. Phe-132 is in the rotated-out conformation, and CE2 is 3.8 A from the dioxindole ring.

### Structure of the TIL Complex with l-Trp and Benzimidazole

**Figure 3:**
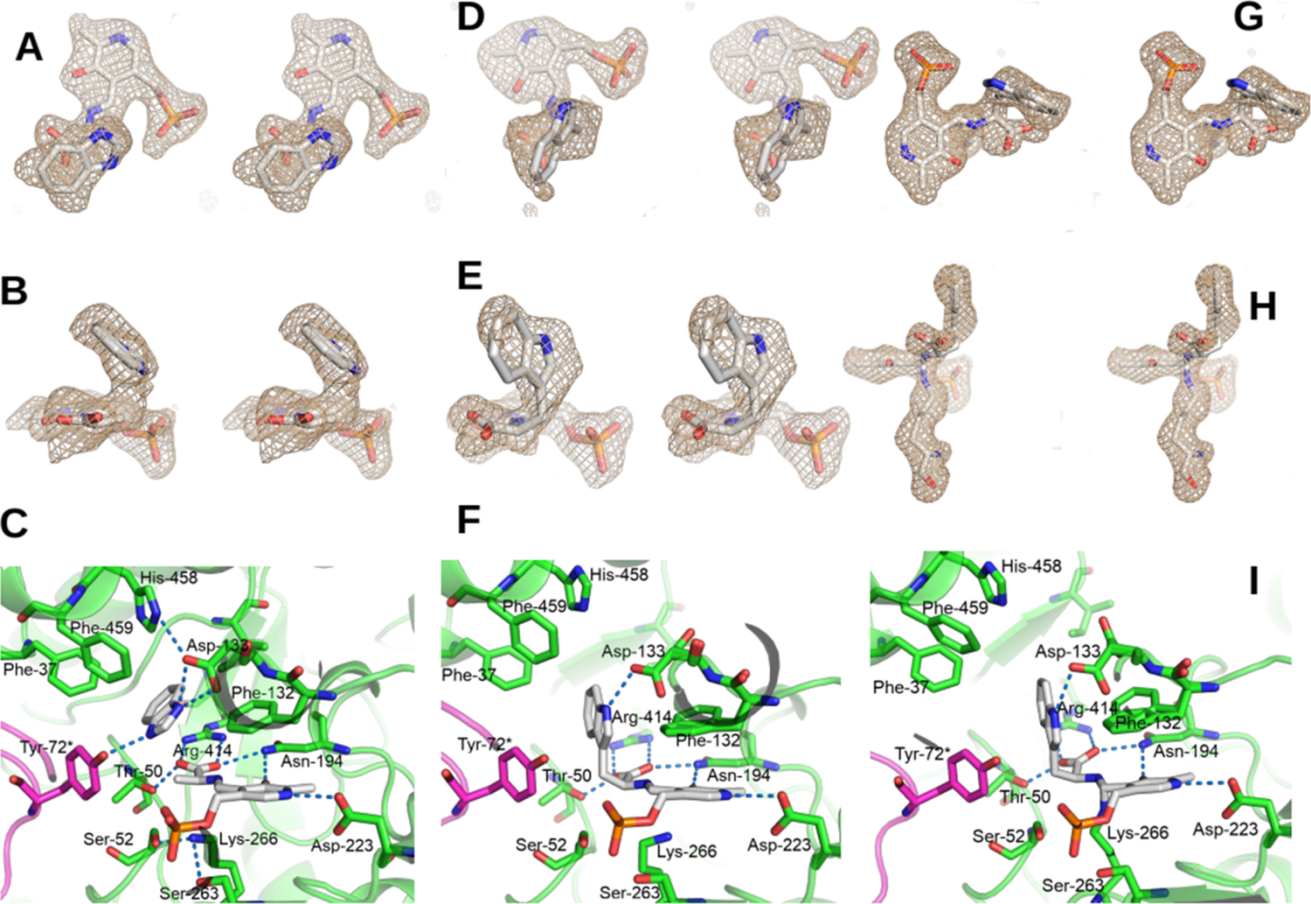
(A) Stereo side view of the sim omit mFo-DFc map at 4σ of the PLP-aminoacrylate-BZI complex in chain A. (B) Stereo top view of the sim omit mFo-DFc map at 4σ of the PLP-aminoacrylate-BZI complex in chain A. (C) View of the environment of the PLP-aminoacrylate-BZI complex in chain A, showing potential hydrogen bonds with blue dashes. (D) Stereo side view of the sim omit mFo-DFc map at 4σ of the PLP-l-Trp aldimine complex in chain B. (E) Stereo top view of the sim omit mFo-DFc map at 4σ of the PLP-l-Trp aldimine complex in chain B. (F) View of the environment of the PLP-l-Trp aldimine complex in chain B, showing potential hydrogen bonds with blue dashes. (G) Stereo side view of the sim omit mFo-DFc map at 4σ of the PLP-l-Trp *gem*-diamine complex in chain C. (H) Stereo top view of the sim omit mFo-DFc map at 4σ of the PLP-l-Trp *gem*-diamine complex in chain C. (I) View of the environment of the PLP-l-Trp *gem*-diamine complex in chain C, showing potential hydrogen bonds with blue dashes. The hydrogen bonds of the phosphate are omitted for clarity.

When TIL is mixed with l-Trp and benzimidazole, a new complex is observed with an absorbance peak at about 345 nm.^17,21,23^ This peak is formed concomitant with the decay of the quinonoid intermediate, with a good isosbestic point, and hence was assigned to the aminoacrylate intermediate. Benzimidazole (BZI) is isosteric and isoelectronic with indole but has no significant π-nucleophilicity, so it can occupy the indole product binding site and stabilize the aminoacrylate complex, allowing its accumulation and observation. Hence, to obtain a structure of an aminoacrylate intermediate, we have now soaked TIL crystals with 50 mM l-Trp and 100 mM BZI. The resulting structure was solved in space group *P*2_1_2_1_2_1_ to a resolution of 1.78 Å with *R*_work_/*R*_free_ of 0.1975/0.2473 (**9BLV**, Table 1). In this structure, only chain A is in a fully closed conformation (Figure S4). This chain contains a PLP complex that does not retain density for the side chain of l-Trp, so it was fit as the PLP-aminoacrylate complex. Above the β-carbon of the aminoacrylate, but not connected by electron density, is additional electron density that fits to the BZI occupying the putative indole product site (Figure 3A,B). The distance from the β-carbon to N1 of the BZI is 3.0 Å, indicating that there is no covalent bond. The aminoacrylate complex has hydrogen bonds to the carboxylate from Arg-414 NH1 (2.7 Å) and NH2 (2.9 Å), the OG of Thr-50 (2.6 Å), and the ND2 of Asn-194 (3.0 Å). ND1 of Asn-194 also donates a hydrogen bond to the 3′-O of PLP (2.9 Å), and N-1 of PLP donates a hydrogen bond to OD2 of Asp-223 (2.8 Å). NZ of Lys-266 donates hydrogen bonds to the OG of Ser-52 (2.3 Å) and Ser-263 (2.9 Å). N-1 of the BZI is hydrogen-bonded to OD1 and OD2 of Asp-133 (2.9 and 3.1 Å, respectively) and OD2 is hydrogen-bonded to NE2 of His-458 (3.2 Å). In addition, N-3 of BZI is hydrogen-bonded to the OH of Tyr-72* (3.0 Å).

Chain B is in an open conformation, and there is electron density attached directly to the β-carbon of the PLP-amino acid complex, but there is no electron density between NZ of Lys-266 and the PLP. Since the ligand carboxylate is not in plane, this density was fit as the external aldimine of PLP and l-Trp (Figure 3D,E). The hydrogen-bonding contacts of the l-Trp external aldimine are very similar to those seen for the external aldimine of 7-aza-l-Trp in Figure 1 (Figure 3F). However, that structure is in a closed conformation, whereas this structure is an open conformation. This shows that the external aldimine exists as an equilibrium mixture of open and closed conformations. Furthermore, Phe-132 and Asp-133 are in a mixture of rotated-in and rotated-out conformations in the l-Trp aldimine, but they are only in the rotated-in conformation for 7-aza-l-Trp. Another difference is that NZ of Lys-266 is not hydrogen-bonded to Ser-52 and Ser-263, but rather is located 3 Å below C4′ of the PLP in the l-Trp aldimine, where it would be immediately after release from the *gem*-diamine.

Chain C is also in an open conformation, but there is clear electron density between NZ of Lys-266 and the C4′ of the PLP (Figure 3G,H), and with N of the bound l-Trp. Thus, this complex was refined as the *gem*-diamine complex of l-Trp and PLP. The NZ-C4′ bond distance was refined to 1.43 Å, consistent with a C–N single bond. The hydrogen-bonding contacts of *gem*-diamine are similar to those of the external aldimine. Phe-132 and Asp-133 are in a mixture of conformations, and the rotated-in conformation of Asp-133 accepts a hydrogen bond from N1 of the l-Trp (3.0 Å). Thus, the critical hydrogen-bonding interactions of the substrate are already formed even in *gem*-diamine, the first covalent intermediate in the reaction mechanism. The distance between the O-3′ of the PLP and the N of the substrate is 2.7 Å, and to the NZ of Lys-266 is 3.0 Å. The substrate N and the O-3′ are nearly in plane with the PLP, indicating that a hydrogen bond is formed, while NZ is located below the PLP plane, in position to leave to form the external aldimine. Finally, Chain D is in an open conformation, with an aminoacrylate complex and BZI bound (not shown). Phe-123 and Asp-133 are in a 40:60 mixture of rotated-in and rotated-out conformations. Similar to chain A, BZI is hydrogen-bonded to Tyr-72* and Asp-133 with N3 and N1, respectively.

### Structure of the TIL Complex with *S*-Ethyl-l-Cys and Benzimidazole

**Figure 4:**
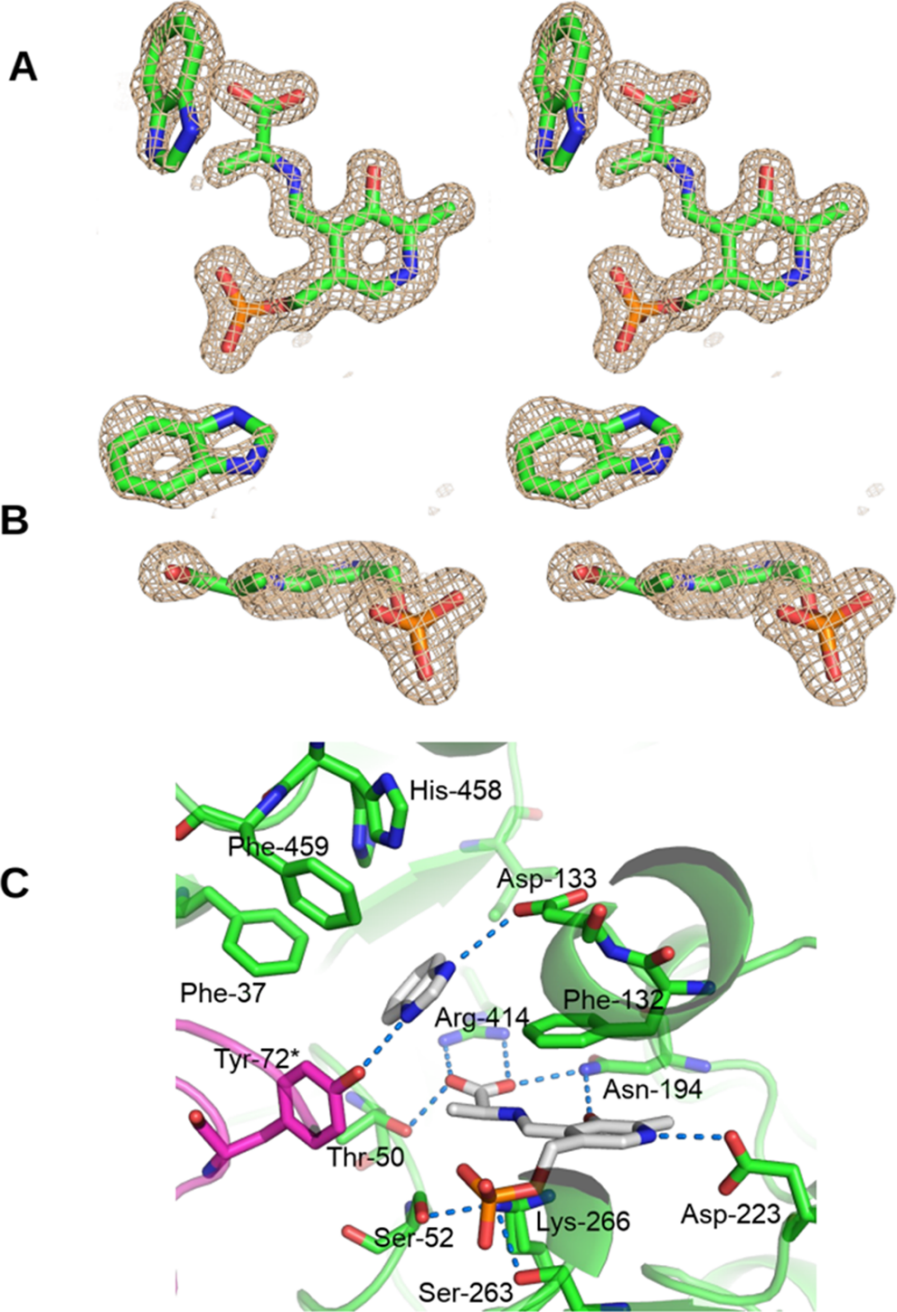
(A) Stereo side view of the sim omit mFo-DFc map at 4σ of the PLP-aminoacrylate-BZI complex in chain B. (B) Stereo top view of the sim omit mFo-DFc map at 4σ of the PLP-aminoacrylate-BZI complex in chain B. (C) Environment of the PLP-aminoacrylate-BZI complex in chain B, showing potential hydrogen bonds with blue dashes. The hydrogen bonds of the phosphate are omitted for clarity.

*S*-Ethyl-l-cysteine (SEC) is an alternative substrate for TIL *in vitro.*(17,24) However, in contrast to the reaction of l-Trp, the elimination reaction of SEC is irreversible. SEC forms a prominent quinonoid peak at 508 nm when mixed with TIL; addition of BZI results in the decay of the quinonoid peak and formation of the 345 nm peak of the aminoacrylate intermediate.^17,24^ We soaked crystals of TIL with 80 mM SEC and 100 mM BZI to obtain a structure of the aminoacrylate intermediate, and we solved the structure of the complex to a resolution of 1.51 Å in space group *P*2_1_2_1_2_1_, with *R*_work_/*R*_free_ = 0.158/0.180 (**9BNJ**, Table 1). Chains A and D are in a 1:1 mixture of open and closed conformations, while chain B is open and chain C is in a closed conformation (Figure S5). None of these chains contain an intact SEC molecule; all four chains contain a PLP-aminoacrylate complex with BZI bound. The open conformation in chain B shows a clear density for the ligands (Figure 4A,B). The hydrogen-bonding contacts of the PLP-aminoacrylate complex are very similar to those of the closed aminoacrylate complex formed from l-Trp, but the BZI binding mode is different. Although Asp-133 is in the rotated-out conformation, it still has a weak hydrogen bond with N1 of the BZI (3.3 Å), and N-3 accepts a hydrogen bond from Tyr-72* (2.7 Å; Figure 4C). The distance from the aminoacrylate β-carbon to N1 of the BZI is 3.8 Å, significantly farther than 3.0 Å in the closed BZI complex. His-458 also shows a mixture of rotamers, one pointing toward Asp-133 and the other rotated away (Figure 4C).

## Discussion

### Conformational Dynamics of TIL

These structures of TIL show equilibrating mixtures of *gem*-diamine, external aldimine, quinonoid, and aminoacrylate complexes, existing in both open and closed active site conformations. The complex of 7-aza-l-Trp has three of four chains in fully closed conformations and one in a 60:40 mixture of open and closed, while that of (3*S*)-DOA has all four chains fully closed. The complex from l-Trp and benzimidazole has only one aminoacrylate complex in a closed conformation and one in a mixture of open and closed, while the *gem*-diamine and external aldimines are open. SEC forms an aminoacrylate and benzimidazole complex in all four chains, with two chains in the open conformation, and two chains that are mixtures of open and closed. The closed conformations move His-458 and Phe-459 into the active site, with the ring of Phe-459 coming into van der Waals contact with the heterocyclic rings in the closed 7-aza-l-Trp, (3*S*)-DOA, and aminoacrylate-BZI complexes.

The side chain of Asp-133 adopts two conformations, rotated either in or out of the active site. The NE2 of His-459 donates a hydrogen bond to OD2 of Asp-133 in the closed complexes of 7-aza-l-Trp and (3*S*)-DOA, where Asp-133 also accepts a hydrogen bond from the NH of the ligand heterocyclic ring (Figures 1C,1F, and 2C). In none of our structures do Asp-133 and His-458 form a hydrogen bond without a hydrogen bond donor ligand in the active site. Thus, a hydrogen bond of Asp-133 with the bound ligand is essential to position it to accept the hydrogen bond from His-458. However, closed conformations of TIL can form without the hydrogen bond, as seen in structures of TIL-l-Ala and TIL-l-ethionine (**8V2K** and **8V4A**, Phillips, R.S., unpublished). With 7-aza-l-Trp and (3*S*)-DOA, Asp-133 is exclusively in the rotated-in conformation and forms hydrogen bonds with His-458. Both OD1 and OD2 of the carboxylate of Asp-133 have similar hydrogen-bonding distances (2.8–3.0 Å) from the N1 of the ligand and N7 of 7-aza-l-Trp. However, only OD2 of the carboxylate accepts a hydrogen bond from NE2 of His-458.

In the closed aminoacrylate complex formed from l-Trp, BZI donates a hydrogen bond from N-1 to Asp-133, which accepts a hydrogen bond from His-458 (Figure 3C), and BZI N-3 also accepts a hydrogen bond from Tyr-72*. This additional hydrogen bond explains why BZI is the best binding indole isostere.^17^ In contrast, the aminoacrylate complex formed from SEC primarily has the BZI bound in an open conformation (Figure 4C), which retains the hydrogen bond from Tyr-72* and to Asp-133, but lacks the hydrogen bond with His-458. This suggests that elimination of ethanethiol from SEC can occur in the open conformation. In this complex, Asp-133 is rotated-out but still forms a weak hydrogen bond to BZI with a longer N–O distance of 3.3 Å. In the open conformation, the distance from N-3 of BZI to the β-carbon of aminoacrylate has increased to 3.8 Å, compared to 3.0 Å for the closed conformation.

The motion of Phe-132 is coupled with that of Asp-133; when the carboxylate of Asp-133 swings in to accept a hydrogen bond from the substrate, the phenyl ring of Phe-132 rotates out about 20° and swings away from the pyridine ring of PLP, forming an approximate perpendicular π–π interaction with the aromatic ring of the substrate. A basic group with a p*K*_a_ of 6.0 was found previously to be essential for the reaction of l-Trp and the binding of oxindolyl-l-Ala, but not for binding of l-Ala, or reaction of an alternative substrate, *S*-methyl-l-Cys, with *E. coli* TIL.^24^ This p*K*_a_ is not seen in the reaction of the H463F variant of *E. coli* TIL with l-Trp.^25^ The D133A variant of *P. vulgaris* TIL has no detectable activity with l-Trp, although it has only about a 2-fold reduction of *k*_cat_ and *k*_cat_/*K*_m_ for SEC.^26^ Furthermore, the H458A variant of *P. vulgaris* TIL has *k*_cat_ only 1.6%, and *k*_cat_/*K*_m_ only 0.4%, with l-Trp, and also loses a p*K*_a_ of 5.3 seen in the pH dependence of *k*_cat_/*K*_m_ of l-Trp for the wild-type enzyme.^26^ In contrast, the reaction of SEC with H458A *P. vulgaris* TIL exhibits *k*_cat_ twice, and *k*_cat_/*K*_m_ 10-fold that of wild-type TIL. Since NE2 of His-458 donates a hydrogen bond to Asp-133 in the closed conformation, both the neutral and protonated imidazole should be capable to form it. Thus, the p*K*_a_ of 5.3/6.0 is most likely that of Asp-133, which must be deprotonated for optimal hydrogen-bonding to both the substrate NH and the NE2 of His-458. The effects of the mutation of His-458 and Asp-133 on the activity with l-Trp, but not SEC, suggest that the hydrogen bond, which requires active site closure, is critical for the catalytic mechanism of l-Trp but does not participate in the reaction of SEC.

### Structure of 7-aza-l-Trp Complexed with TIL

7-Aza-l-Trp was used to complex with TIL crystals because it is a very slow substrate (*k*_cat_ ∼ 1% that of l-Trp) that was shown in previous rapid-scanning stopped-flow spectroscopic experiments to form equilibrating mixtures of external aldimine and quinonoid complexes at rates comparable to l-Trp.^22,23^ Surprisingly, we found that the quinonoid structures of 7-aza-l-Trp with imposed planar restraints for the azaindole ring did not refine well; the bond angles of Cα-Cβ-C-3 and the PLP ring had significant distortion (>4–6 σ) from the optimized values based on the restraints. The observed Cα–Cβ–C3 bond angle of the ligand was 92° vs 112° predicted, and C5–C4–C4′ (135° observed vs 122° predicted), and C3–C4–C4′ of the PLP ring also showed severe distortions (106° observed vs 119° predicted). In contrast, when the azaindole ring was modeled with restraints calculated for a tautomeric azaindolenine ring, with tetrahedral rather than trigonal geometry at C3 of the ring, refinement did not result in significant deviations from the predicted bond angles either for the ligand or PLP. This strained geometry in the azaindole ring is imposed partially by Phe-459 upon quinonoid intermediate formation in the closed conformation. If the pyrrole ring of azaindole would remain planar in the quinonoid complex, there would be a severe clash between the substrate and the phenyl ring of Phe-459, with calculated distances of 1.6 Å from CZ and 2.0 Å from CE1 to N-7, compared to 3.1 Å from CZ of Phe-459 in the bent structure. In contrast, the bent structure can have attractive perpendicular π–π interactions between Phe-132 and Phe-459 with the substrate ring. Furthermore, the hydrogen bond of the azaindole NH with Asp-133 would be broken if the ring remains planar in the quinonoid complex, and there can be OH–π hydrogen-bonding of C3 with the OH of Tyr-72*, since there would be a partial negative charge on the partially pyramidalized C3 in the bent geometry. The net effect of these interactions is to stabilize the bent substrate geometry relative to the planar ring.

Although the refinement restraints were calculated based on the azaindolenine tautomer, the refinement did not use explicit hydrogens, and we do not believe that the azaindole ring has actually been protonated on C3 in the quinonoid structures. C3 of the 7-azaindole ring is expected to be much less basic on C3 than indole (p*K*_a_ ≈ −3.5^27^) due to the electron-withdrawing influence of the N7 nitrogen in the pyridine ring. Previous calculations on the gas-phase proton affinity of C3 of 7-azaindole showed that it is reduced by 10 kcal/mol (ca. 7 pH units) compared to indole.^28^ We repeated these calculations with a higher level of theory (cc-DLNPO), and we found that the gas-phase proton affinity of 3-methyl-7-azaindole, a better model for 7-aza-l-Trp, at C3 is about 5 kcal/mol (ca. 3.6 pH units) less than that of 3-methylindole. The torsion angle for Cβ–C3–C4′–C4 of 7-aza-l-Trp in the quinonoid complex is about 40°, while the corresponding torsion angle in the external aldimine is about 1° (Table 2). Similarly, we found previously that 3-fluoro-l-tyrosine bound to the inactive Y71F variant of tyrosine phenol-lyase (TPL), a closely related enzyme mechanistically, also shows a closed conformation, with a bent geometry for the phenol ring, up to 27° out of plane, giving an estimated 12–21 kcal/mol of strain energy.^29^

**Table 2:**
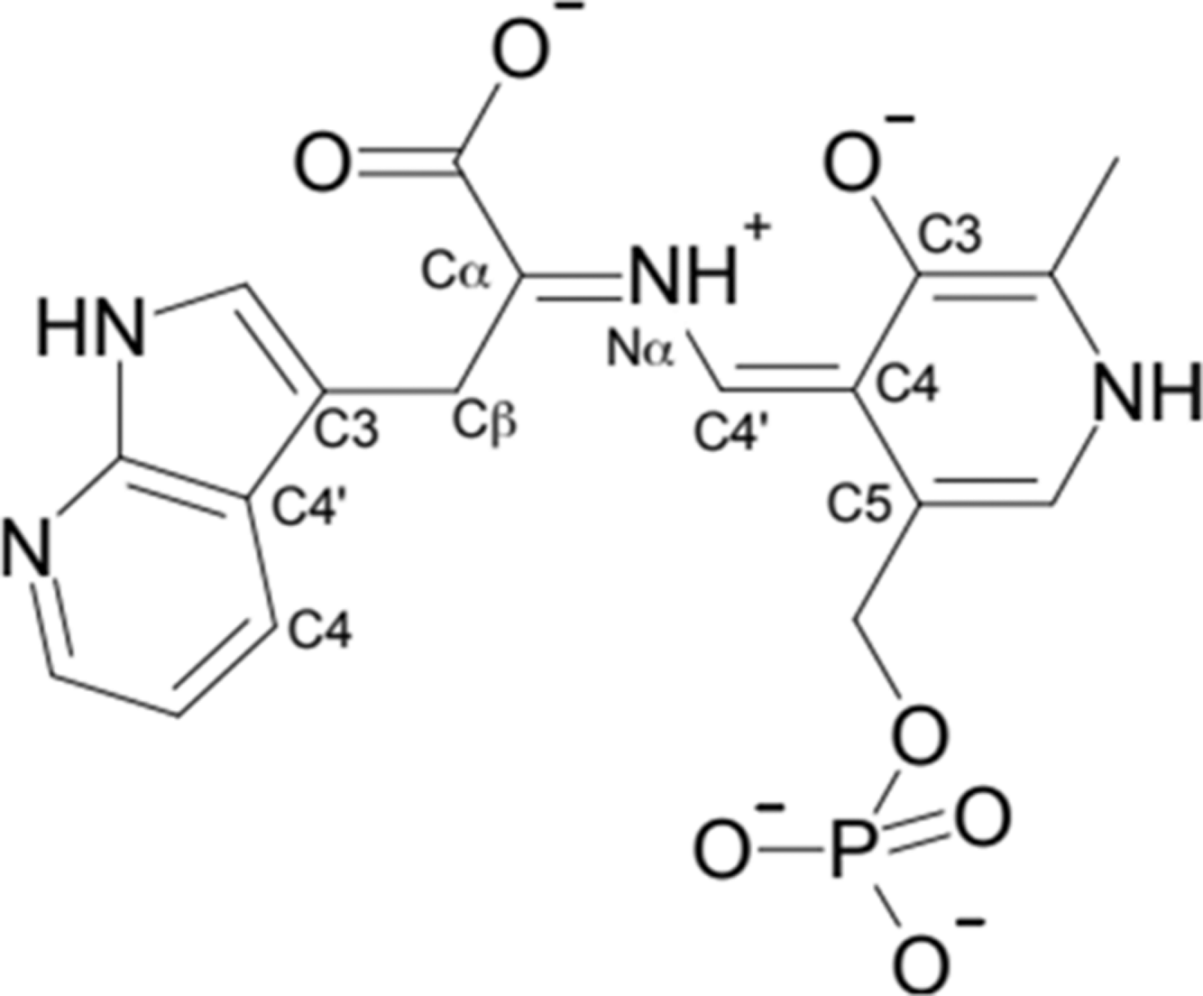
Key Bond Angles in TIL-7-aza-l-tryptophan Structures

angle	chain			
	A (deg)	B (deg)	C (deg)	D (deg)
Cα–Cβ–C3	111.6	117.4	114.1	117.8
Cβ–C3–C4′–C4	0.9	41.5	1.5	43.9

In previous studies, we concluded that proton transfer is rate-determining for the reaction of aza-tryptophans, since quinonoid intermediates form at a rate comparable to l-Trp, but BZI does not stabilize an aminoacrylate intermediate.^23^ The subsequent rate of release of iminopyruvate from the aminoacrylate intermediate should be the same for all substrates irrespective of the leaving group. As discussed above, the proton affinity of the 7-azaindole ring is reduced compared to indole, resulting in a lower p*K*_a_. Although gas-phase proton affinities are modulated in solution, the relative proton affinities for the isoelectronic ring systems should be comparable. The reduced proton affinity would be expected to result in a slower reaction if the proton transfer is rate-limiting. In contrast, if proton transfer to C3 had already occurred, the lower p*K*_a_ of 7-azaindole would make it a better leaving group; hence, elimination should be as fast or faster than indole, and an indolenine intermediate should be less, not more, stable than that of l-Trp. We determined previously from rapid quench experiments that elimination of indole for wild-type *E. coli* TIL occurs in a burst, with *k* ∼ 30 s^–1^, significantly faster than the steady-state *k*_cat_ = 4 s^–1^,^29^ indicating that both elimination and product release are partially rate-determining. Multiple isotope effects on the formation of the aminoacrylate intermediate from l-Trp, followed directly by stopped-flow spectrophotometry, suggested that proton transfer to C3 of the indole and Cβ–C3 bond cleavage is concerted rather than stepwise, since the primary isotope effects of α-deuteration, solvent isotope effects, and secondary isotope effects of β-deuteration are additive.^21^ Thus, the elimination reaction of 7-aza-l-Trp is intrinsically 30/0.04 = 750-fold slower than that of l-Trp. This corresponds to a reasonable Bronsted β = 0.8, assuming a p*K*_a_ difference of 3.6, consistent with rate-determining proton transfer for 7-aza-l-Trp. We note that OD2 of Asp-133 is only 2.9 Å from N7 of the azaindole in chain B, suggesting that there could be a hydrogen bond. N7 is the most basic site in 7-azaindole, with a p*K*_a_ of 4.59 in solution,^28^ so it is not surprising that it could be protonated in the active site. If N7 of 7-aza-l-Trp is protonated in the complex with TIL, then the basicity of C3 is expected to be decreased even more compared to indole since a dicationic indolenine would then be formed.

### Reaction Mechanism of TIL

The proposed reaction mechanism of the TIL, as shown in Scheme 1, does not include conformational dynamics. PLP-dependent enzymes provide unique visible spectroscopic features for each covalent intermediate in the reaction mechanism. Rapid-scanning stopped-flow spectrophotometry has provided evidence previously for the formation of external aldimine, quinonoid, and aminoacrylate intermediates in the reaction of TIL with l-Trp.^17,21,23,30^

**Figure 5:**
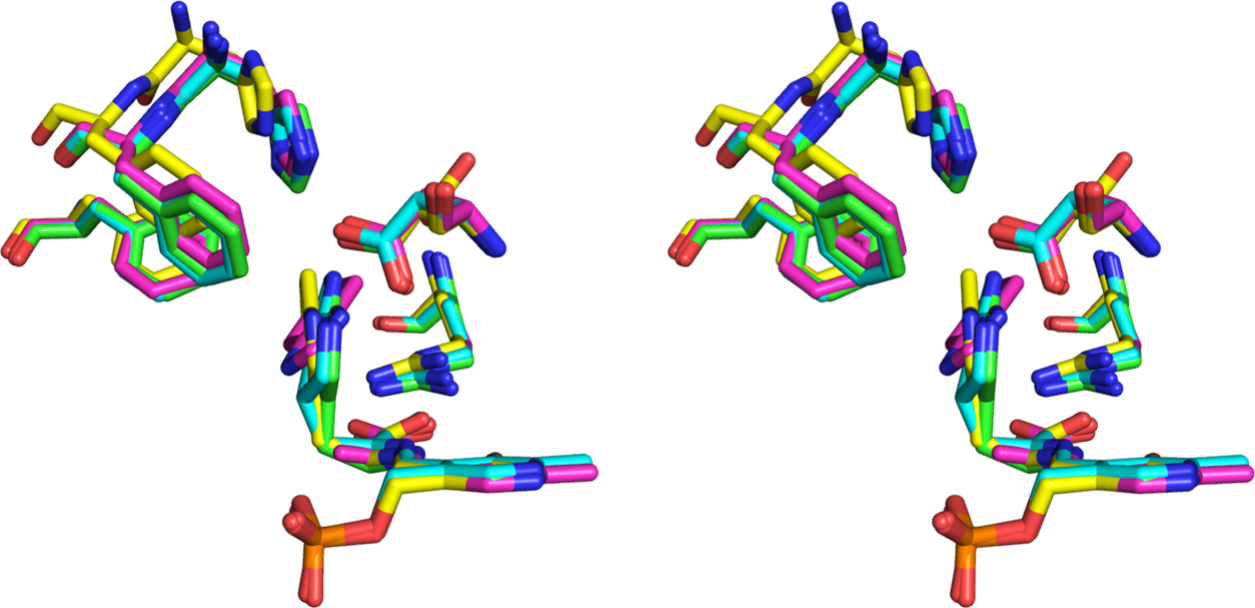
Stereo view of the overlay of the structures of the open *gem*-diamine of l-Trp (yellow), the closed external aldimine of 7-aza-l-Trp (green), the quinonoid complex of 7-aza-l-Trp (cyan), and the closed aminoacrylate-BZI complex (magenta). Phe-37, Asp-133, Arg-414, His-458, and Phe-459 are also shown.

The rapid kinetics also provided evidence for a conformational change in the quinonoid intermediate coupled with elimination.^23^ In the present study, we have obtained crystal structures of all of the key intermediates in the proposed reaction mechanism of TIL for the first time. In the overlay of the open and closed external aldimine, closed quinonoid and closed aminoacrylate structures (Figure 5), we can see the motion of the substrate during the catalytic cycle. The hydrogen bond of the substrate with Asp-133 forms in the early *gem*-diamine complex in the open conformation (yellow structure). Forming the external aldimine in the closed conformation results in a small movement of the substrate ring toward Asp-133 and formation of a hydrogen bond with His-458 (green structure). Quinonoid intermediate formation then results in the substrate with the ring bent about 40° (cyan structure); elimination of the indole leaving group results from rotation of the ring by another 36° and movement of C3 about 1 Å from the β-carbon to break the Cβ–C3 bond and give the closed indole aminoacrylate complex (magenta structure). Thus, the bent geometry introduced in the ring of the quinonoid intermediate gives the bound substrate a geometry intermediate between the reactant state and transition state, moving the substrate up the reaction coordinate and reducing the activation energy. This energy required to achieve the bent geometry is then relieved as the transition state for elimination is reached.

**Scheme 3:**
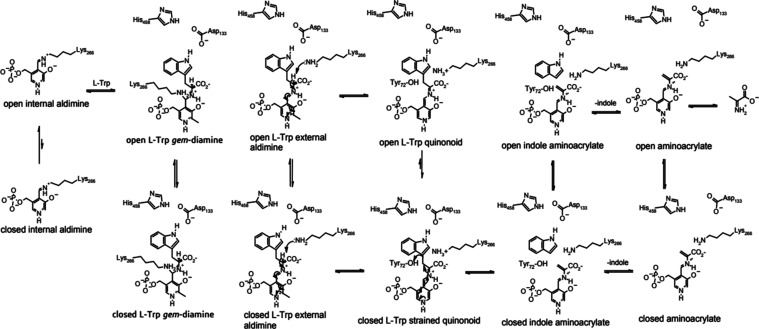
Mechanism of TIL Including Conformational Dynamics

The revised mechanism of TIL, including conformational dynamics, consistent with these new structural data, is shown in Scheme 3. In the resting state, TIL exists primarily as the open conformation. l-Trp, or another amino acid, initially binds to the open conformation of TIL to form *gem*-aldimine in an open conformation (Figure 3I). The O-3′ of the PLP is equidistant from NZ of Lys-266 and Nα of l-Trp, so it is likely that O-3′ shuttles a proton between them to facilitate the transaldimination. Release of Lys-266 from the *gem*-diamine forms the external aldimine in the open conformation (Figure 3F), which is now in equilibrium with the closed conformation (Figure 1C). Deprotonation of Cα by Lys-266 can now occur to form the quinonoid complex of l-Trp, in closed conformation due to the hydrogen bond with Asp-133 and His-458 (Figure 1F). Although the electron density maps show that NZ of Lys-266 in the external aldimine is hydrogen-bonded to OG of Ser-51 and Ser-263, 3.9 Å away from Cα, and is not in a suitable geometry for proton transfer, it has an alternative allowed conformation (“mmtm”), which puts NZ directly in line with Cα, and only 3.0 Å away, in an ideal position to abstract the α-proton (Figure S6). There is no observed electron density that fits this conformation of Lys-266, so it must be a structure that only exists transiently during deprotonation/reprotonation of Cα. We note that there are a wide range of rate constants observed for quinonoid intermediate formation, depending on the side-chain structure, from ∼1 s^–1^ for l-Ala, ∼20 s^–1^ for l-ethionine, ∼100 s^–1^ for 7-aza-l-Trp, and >100 s^–1^ for l-Trp.^34^

What is the trigger to shift the equilibrium position farther toward the closed conformation in the quinonoid complex? It is not simply the side chain of the substrate forming the hydrogen bond with His-458, through Asp-133, since both l-Ala and l-ethionine can form quinonoid intermediates with closed conformations without forming the hydrogen bond with Asp-133 (**8V2K** and **8V4A**, Phillips, R.S., unpublished). Mutation of His-458 to Ala in TIL resulted in an enzyme with only 1.6% activity with l-Trp,^26^ but without affecting binding of amino acids and quinonoid intermediate formation. However, we found that the effects of temperature and pressure on quinonoid intermediate formation from l-Trp and l-Met by the H463F variant of *E. coli* TIL suggest that histidine is important for preorganization of the substrate for reaction.^31,32^ This preorganization could be due to the closed conformation stabilized by hydrogen-bonding of Asp-133 and His-458.

Arg-414 forms a salt bridge with the ligand α-carboxylate in the external aldimine and undergoes a small movement (∼1 Å) as the carboxylate moves from tetrahedral geometry in the external aldimine to a planar geometry in the quinonoid complex (Figure 5). Arg-414 is located at the pivot point of the extended β-turn from residues 389–414, so this small movement of Arg-414 may result in the movement of the loop and be amplified into the much larger movement of the small domain in the closed conformation. Consistent with this hypothesis, limited trypsin proteolysis at Lys-406 of the flexible loop of *E. coli* TIL results in an inactive enzyme with altered conformational properties.^33^

Structural analogues that mimic the strained indole ring of the closed l-Trp quinonoid complex, such as oxindolyl-l-alanine and (3*S*)-DOA, are competitive inhibitors, with *K*_i_ values of 1–10 μM that form stable quinonoid complexes exclusively in closed conformations. The bent 7-azaindole ring overlays very well with the rings of oxindolyl-l-alanine and (3*S*)-DOA, which have been considered previously as transition-state analogues for TIL.^18,19^ A compound similar to (3*S*)-DOA, (3*S*)-3-chlorooxindolyl-l-alanine, has been reported recently as an inhibitor of TIL for potential treatment of chronic kidney disease.^13^ We now confirm structurally that these compounds resemble the geometry of the quinonoid intermediate of l-Trp leading to the transition state. It is interesting that the *K*_m_ for 7-aza-l-Trp is 5 mM,^23^ about 25-fold higher than that of l-Trp, suggesting that binding is weaker despite very similar binding contacts of the respective external aldimines (compare Figures 1C and 3F). This suggests that some of the binding energy is used to stabilize the distorted geometry of the quinonoid complex of 7-aza-l-Trp.

**Figure 6:**
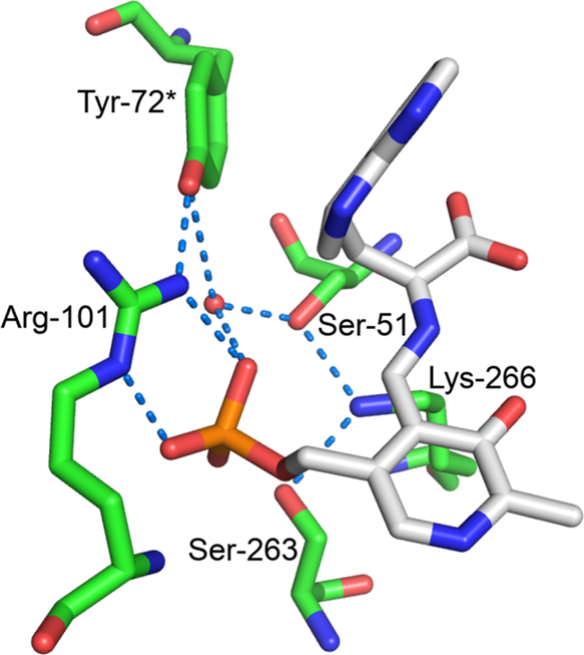
View of the hydrogen-bonding network of Lys-266 in the 7-aza-l-Trp quinonoid complex.

Concomitant with proton transfer from OH of Tyr-72* to C3, the elimination of indole takes place to give the closed aminoacrylate intermediate, with indole noncovalently bound by a hydrogen bond to Asp-133, similar to that of BZI (Figure 3C). Furthermore, the hydrogen bond of the indole N1 with Asp-133 can also facilitate proton transfer by stabilizing a transient partial positive charge on N-1 in the transition state. Consistent with this idea, mutation of Asp-133 to Ala resulted in an inactive enzyme for l-Trp, although it retained activity with other substrates with good leaving groups.^26^ NZ of Lys-266 is connected to OH of Tyr-72* by a network of hydrogen bonds involving conserved Ser-51, a phosphate oxygen, and a water (Figure 6), which may allow concomitant deprotonation of Lys-266 by a Grötthus-type mechanism with proton transfer from Tyr-72* during elimination, so that it can be available as the free base to form a *gem*-diamine for the subsequent iminopyruvate release step.

This is consistent with the observed isotope effect on aminoacrylate intermediate formation with α-^2^H-l-Trp,^21^ since the proton originating on Cα has been transferred to NZ of Lys-266. Furthermore, there is observed small internal return (<7.9%) of the α-proton to C3 of the indole leaving group,^34^ suggesting that the α-proton may be retained in these hydrogen bonds, eventually transferred to Tyr-72*, and from there to the indole leaving group, albeit in multiple turnovers. However, we did not find evidence for internal return of the α-proton of l-Trp to indole by gas chromatography-mass spectrometry (GC-MS) in single-turnover rapid quench experiments with l-Trp (Phillips, R.S., unpublished). Indole may be released from the closed aminoacrylate conformation, since indole is nonpolar and lipophilic, or after opening the active site to give the open aminoacrylate intermediate, which we observed with the SEC soaked crystals (Figure 4C). Finally, nucleophilic attack of Lys-266 on the Schiff’s base of the aminoacrylate forms a *gem*-diamine (not shown). Although we did not observe a structure of an aminoacrylate *gem*-diamine for TIL, we did see this intermediate in the reaction of TPL with l-Ser.^35^ The aminoacrylate *gem*-diamine is now nucleophilic and can be protonated on the β-carbon, followed by the release of iminopyruvate^36^ and restoration of the open internal aldimine to perform another catalytic reaction. Protonation of the aminoacrylate on Cβ is stereospecific, occurring on the same face of the alkene as the elimination of the leaving group, resulting in a chiral methyl group if chiral β-tritiated l-Trp or l-Ser is used in D_2_O.^34^ We note that the stereochemistry of this β-elimination reaction is *anti*, with the base and the leaving group on opposite sides of the substrate; thus, Lys-266 cannot be the proton donor to Cβ, since it is on the opposite face of the aminoacrylate. There is no other suitable proton donor other than Tyr-72* on the top face of the aminoacrylate. However, it is more than 4 Å from the aminoacrylate structures. Possibly, protonation of Cβ of the aminacrylate is mediated by water, hydrogen-bonded to Tyr-72*.

### Implications for Enzyme Catalysis

The Pauling hypothesis proposes that transition-state stabilization is the major contribution to the rate acceleration of enzymatic reactions.^37^ However, there have long been suggestions that ground-state effects, often called “ground-state destabilization”, may also play a role.^38^ For example, enzymes may bind the substrate in a high energy-reactive conformation. Bruice called these conformations “Near Attack Conformations” or NACs.^39^ These higher-energy structures should be observable in X-ray crystal structures of enzymes with bound ligands. Atomic resolution X-ray crystal structures of transketolase show clear evidence for bond length and angle distortion of the covalent thiamine-substrate complex to activate the substrate for the reaction.^40^ Hyperconjugation was shown to weaken the α–C–H bond of the external aldimine of aspartate aminotransferase, measured by equilibrium isotope effects, that facilitates deprotonation.^41^

Do other enzymes in addition to TIL show distortion of the aromatic rings of substrates related to catalysis? Interestingly, the high-resolution X-ray structure of 5-carboxyvanillate decarboxylase bound to a nitro analogue shows the nitro group bent 23° out of plane of the aromatic ring.^42^ This was proposed to decrease aromaticity in the ring and increase basicity at the carbon bearing the carboxylate group, allowing *ipso* ring protonation prior to decarboxylation. We showed previously that TPL, an enzyme structurally and mechanistically related to TIL, shows clear evidence for ground-state strain in catalysis.^35,43,44^ The aromatic ring of the tyrosine substrate is bent out of plane with the Cβ-Cγ bond by about 20–27°, corresponding to 12–20 kcal/mol of strain energy, in the crystal structures of the Y71F and F449H variants, which have *k*_cat_ values reduced as much as 10^4^-fold.^29^ Similar to TIL, distortion of the substrate is initiated by closing the active site, bringing Phe-448 and Phe-449 into van der Waals contact with the phenyl ring of the substrate. Mutagenesis of Phe-448 and Phe-449 to alanine suggests that the ground-state strain may contribute up to 10^8^ to the catalytic rate acceleration.^43,44^ Similar experiments with the mutagenesis of Phe-459 of TIL are planned. The bent aromatic ring of the TPL substrate is stabilized by forming new hydrogen bonds of the OH with OG of Thr-124 and NH2 of Arg-381. Only a small movement, ∼ 1 Å, of C1 of the bent phenol ring results in the product phenol-aminoacrylate complex.^35^ The effects of pressure, temperature, and heavy enzyme kinetic isotope effects on the reaction of TPL suggest that the conformational change is kinetically coupled with the elimination chemistry.^35^

## Conclusions

Conformational dynamics plays a critical role in the mechanism of TIL. Substrates bind to an open conformation of the enzyme to form *gem*-diamine and then external aldimine intermediates, which are in equilibrium with closed conformations. The closed conformation brings catalytically essential Asp-133 into the active site to bridge between N1 of the substrate heterocyclic ring and NE2 of His-458. Upon quinonoid intermediate formation, this conformational change results in bending of the substrate, facilitating proton transfer from Tyr-72* to C3 and concerted C3–Cβ carbon bond cleavage. The closed indole aminoacrylate intermediate opens to release indole and iminopyruvate to complete the catalytic cycle. The role of conformational dynamics as well as the chemical reaction mechanism should be considered in the rational design of inhibitors of TIL that could be useful as drugs, for example, for chronic kidney disease.^13^

## Methods

### Materials

7-Aza-L-tryptophan was prepared by the reaction of 7-azaindole with l-serine catalyzed by tryptophan synthase.^45^ (3*S*)-Dioxindolyl-l-alanine was prepared as described by Labroo and Cohen.^46^ Other materials were obtained from standard commercial sources.

### Enzyme Purification and Crystallization

TIL from *P. vulgaris* was expressed in *E. coli* BL21(DE3) tn5:tnaA and purified as described previously.^20^ Crystallization was performed using a modification of previously published conditions.^47^ The protein (2 μL, 20 mg/mL) in 0.1 M potassium phosphate, pH 8.0, 1 mM dithiothreitol (DTT), and 0.1 mM PLP was mixed 1:1 with the same buffer containing 0.2 M CsCl or 0.2 M KCl and 22% PEG 4000. The crystals form in a variety of morphologies, including hexagonal rods, prisms, and trapezoidal plates. We found that the plates, although rather thin in one dimension, consistently showed higher resolution in diffraction data and were used exclusively for the work reported herein. The crystallization conditions above were optimized to obtain more of the plates.

### Complexation and Data Collection

The crystals were transferred to a cryosolution containing 0.1 M potassium phosphate, pH 8.0, 1 mM DTT, 0.1 mM PLP, 10% (v/v) of a 1:1:1 mixture of dimethylsulfoxide (DMSO), glycerol, and ethylene glycol, either l-Trp (50 mM) and BZI (100 mM), *S*-Et-l-Cys (60 mM) and BZI (100 mM), 7-aza-L-tryptophan (50 mM), or (3*S*)-dioxindolyl-l-alanine (10 mM), 0.1 M sodium ascorbate, and 25% PEG 4000. The crystals were flash frozen in liquid N_2_ after incubation for 1–2 min. Longer incubations resulted in a progressive loss of resolution. Data were collected at 100 K on beamlines BM-22 and ID-22 at SER-CAT and ID-19 at NSLS2.

### Structure Determination

The data were indexed and integrated with XDS^48^ and AutoPROC,^49^ using AIMLESS^50^ for scaling. Despite their similar morphology, these crystals were found to adopt multiple space groups, either *P*2_1_, *P*2_1_2_1_2, or *P*2_1_2_1_2_1_. The assignment of the correct space group was confirmed with LABELIT.^51^ Some of the crystals also show anisotropy, so the data were processed, and ellipsoidal resolution limits were determined with STARANISO,^52^ using the standard resolution local cutoff of *I*/σ of 1.2. STARANISO analyzes the anisotropy of the data and applies Baseyian statistics to estimate the amplitudes. This improves the data in the weaker diffracting direction and thus extends the resolution limits. The output from STARANISO was then used for phasing by molecular replacement with 5W19.pdb using PHASER^53^ in PHENIX.^54^ Model building was performed with COOT^55^ and refinement with PHENIX.refine.^56^ The ligands were omitted, and simulated annealing was performed during the initial refinement cycles to avoid model bias. The ligand structures were created in Gaussview (Gaussian, Inc.), and geometry was optimized with PM6, then exported as mol2 files. The ligand cif files were then created with eLBOW^57^ in PHENIX. The figures were prepared with the open source version of PYMOL (The PyMOL Molecular Graphics System, Version 2.5 Schrödinger, LLC.)

### Enzyme Inhibition

Inhibition of TIL by (3*S*)-dioxindolyl-l-alanine was performed using SOPC as the substrate, following the absorbance decrease at 370 nm (Δε = −1860 M^–1^ cm^–1^).^58^ The concentration of SOPC was varied between 20 and 200 μM with 0, 10, and 20 μM (3*S*)-dioxindolyl-l-alanine. The *K*_i_ was determined by fitting the data to eq 2 with COMPO^59^
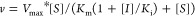
2

### Computation

Computations were performed with ORCA 5.0.1,^60^ using ChimeraX^61^ as a graphical interface with the SEQCROW tool.^62^ The structures of 3-methylindole and 3-methy-7-azaindole were optimized with B3LYP with a double-ζ basis set and then the single-point energies calculated at the coupled cluster level with DLNPO, using a triple-ζ basis set. The C-3 protonated structures were then created and optimized, and energies were calculated to obtain proton affinities from the energy differences.

## Supporting Information Available

Comparison of the structures of TIL complexed with 7-aza-l-Trp, l-Trp + benzimidazole, (3*S*)-dioxindolyl-l-alanine, and *S*-ethyl-l-cysteine + benzimidazole (PDF)
