# Supplementary material for: Structural Snapshots of Proteus vulgaris Tryptophan Indole-Lyase Reveal Insights into the Catalytic Mechanism
Source: ACS Catal. 2024 Jul 18;14(15):11498–511. doi: 10.1021/acscatal.4c03232 (PMC11301627; doi:10.1021/acscatal.4c03232)
Supplement: Supplementary file 1 — cs4c03232_si_001.pdf [file cs4c03232_si_001.pdf]

Structural Snapshots of *Proteus vulgaris* Tryptophan Indole-lyase Reveal Insights into the Catalytic Mechanism

Robert S. Phillips[‡§]*, S. Meredith Brown[§], and Ravi S. Patel[§]

[‡]Departments of Chemistry, University of Georgia, Athens, GA 30602

[§]Department of Biochemistry and Molecular Biology, University of Georgia, Athens, GA 30602

*To whom correspondence should be addressed at Department of Chemistry, University of Georgia, Athens, GA 30602, E-mail: plp@uga.edu

Supporting information

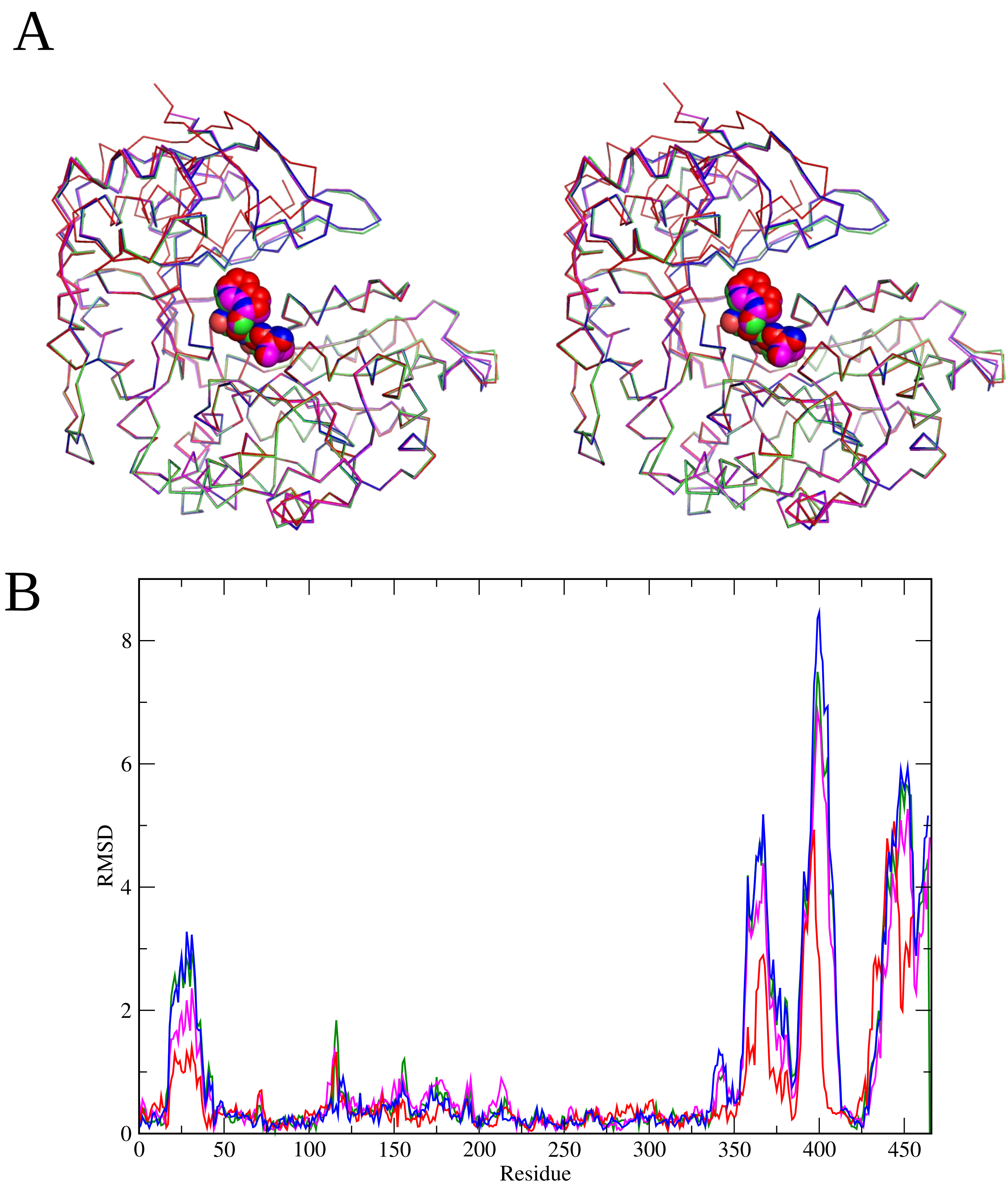

Figure S1. A. Overlay of the Cα backbones of chains of the TIL-7-aza-L-tryptophan complex. Green: Chain A; magenta: Chain B; red: Chain C; blue: Chain D. B. RMSDs of the TIL-7-aza-L-tryptophan complex relative to the unliganded enzyme. The chain colors are as in A.

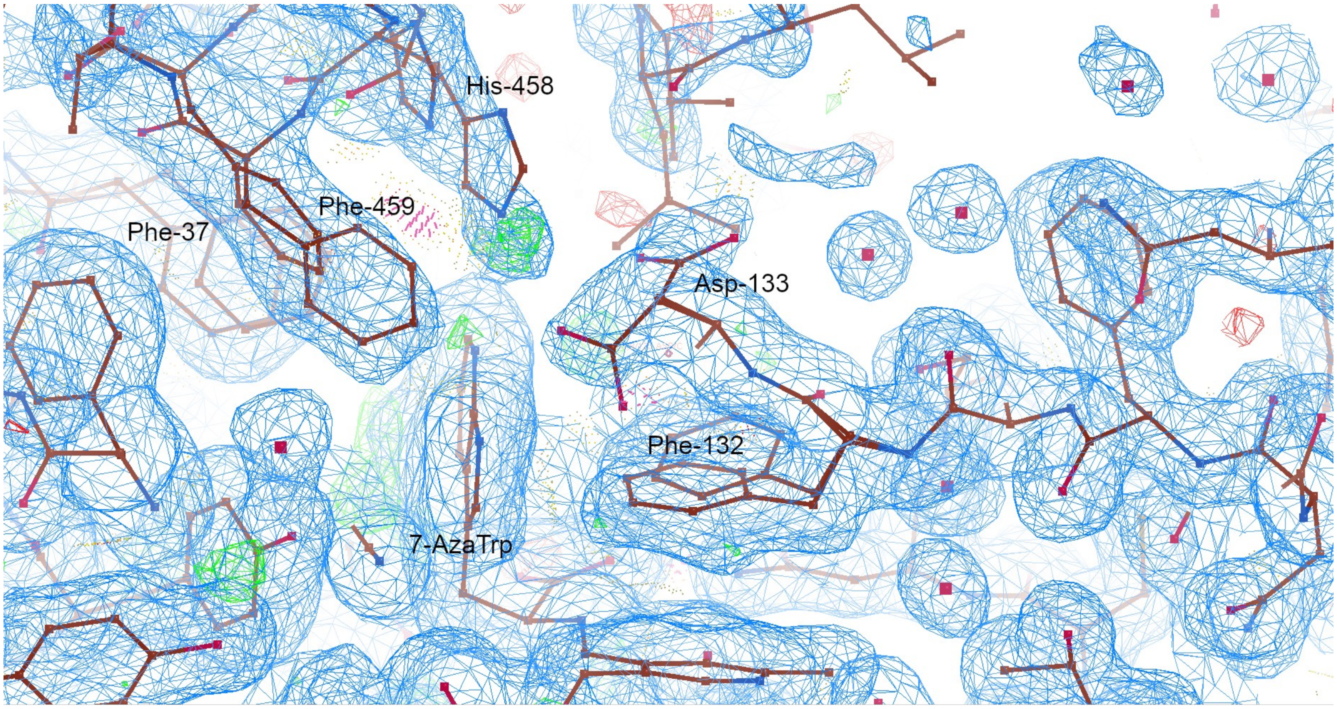

Figure S2. 2mFo-DFc map of chain C of the TIL complex with 7-aza-L-tryptophan at 0.5 σ, showing the alternate conformations of Phe-37, Phe-132, Asp-133, His-458, and Phe-459.

A

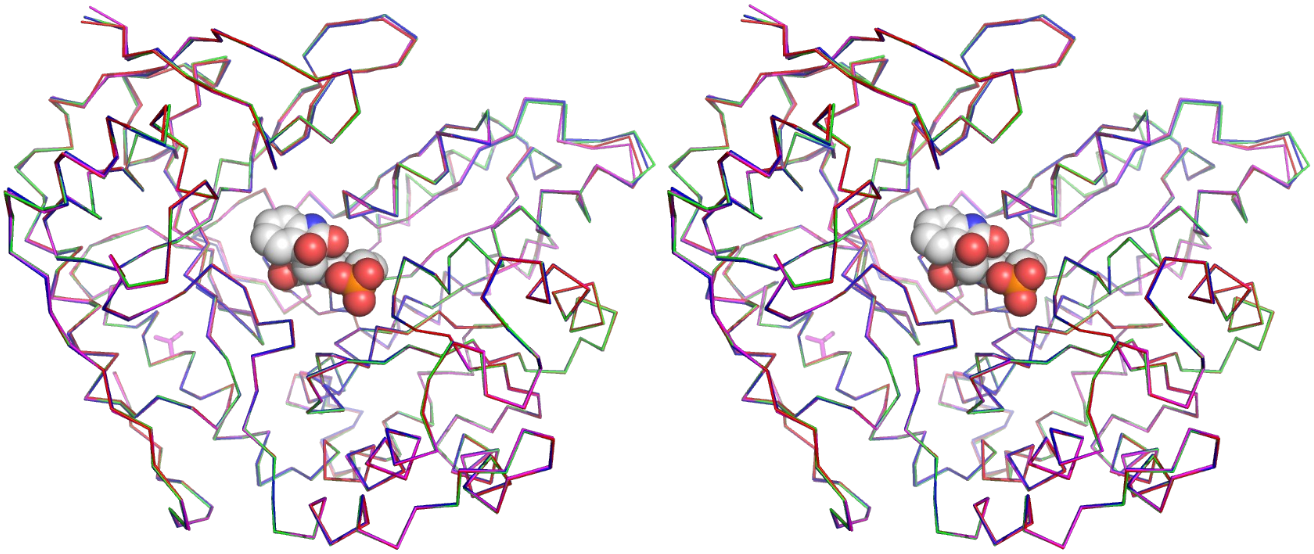

B

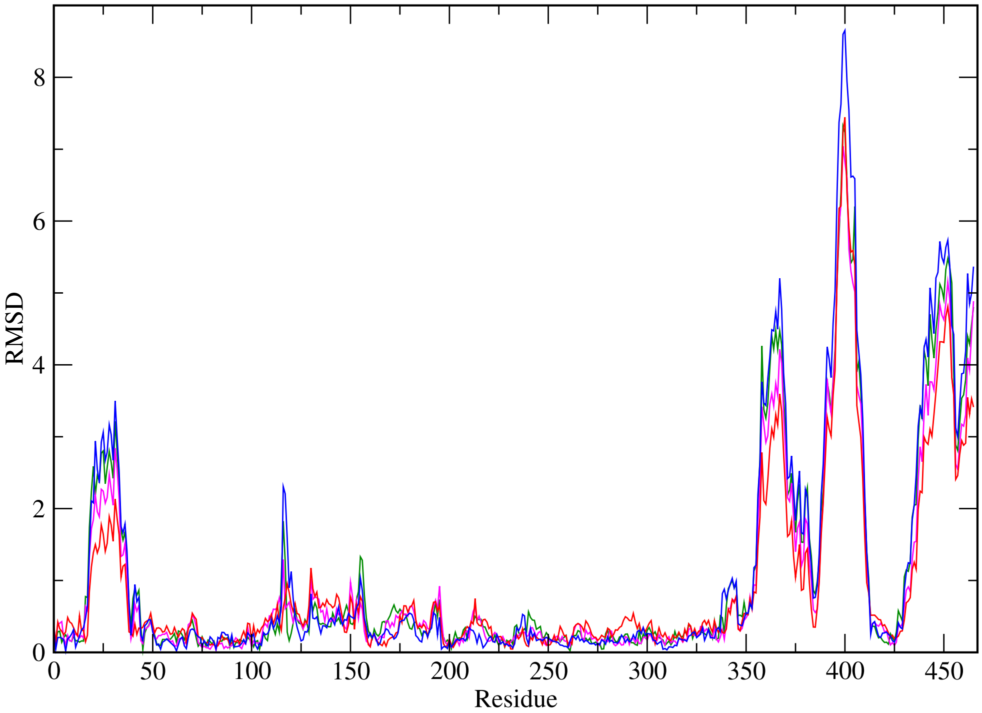

Figure S3 A. Overlay of the Cα backbones of chains of the TIL-(3*S*)-dioxindolyl-L-alanine complex. Green: Chain A; magenta: Chain B; red: Chain C; blue: Chain D. B. RMSDs of the TIL-(3*S*)-dioxindolyl-L-alanine complex relative to the unliganded enzyme. The chain colors are as in A.

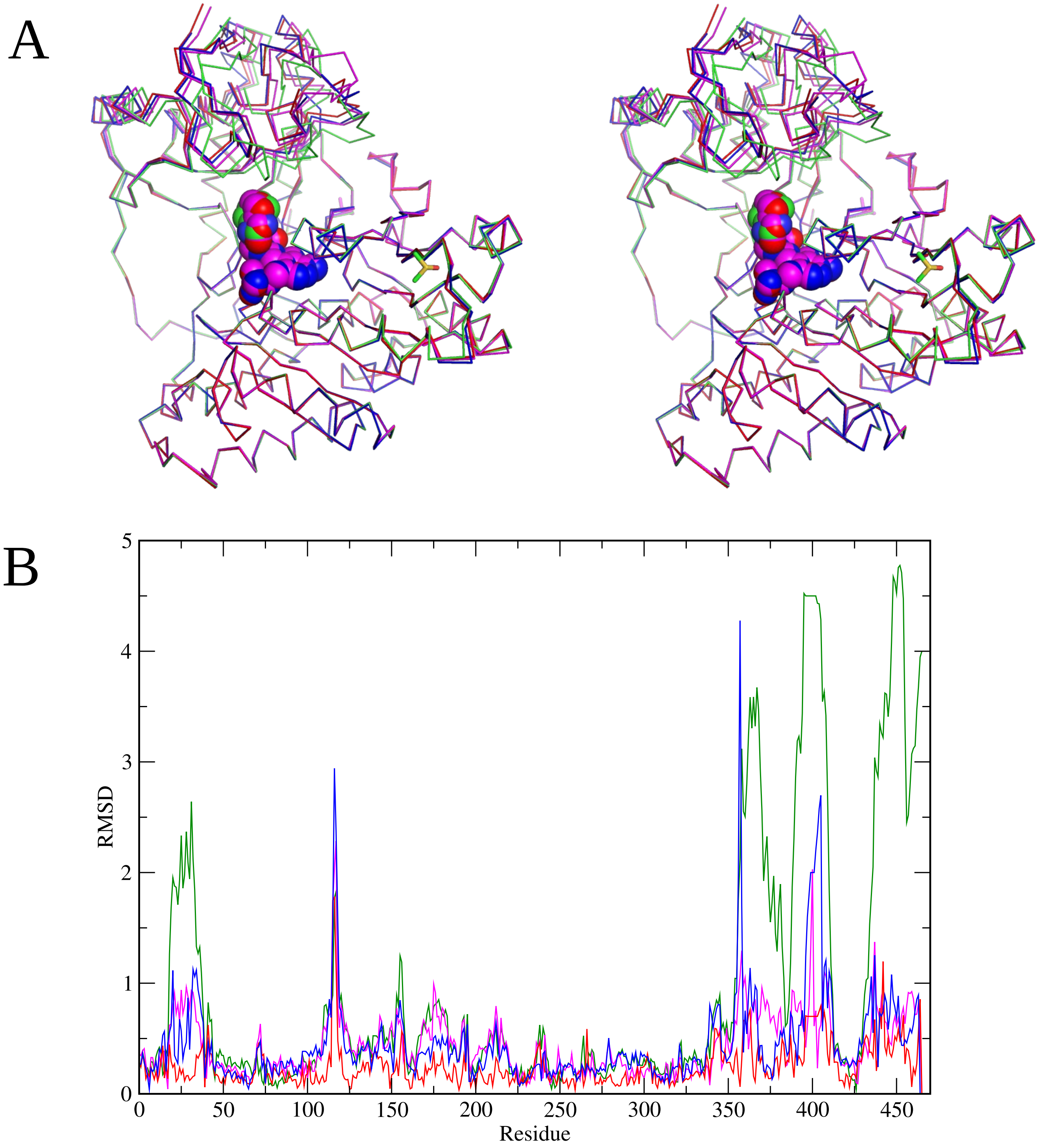

Figure S4 A. Overlay of the Cα backbones of chains of the TIL-L-Trp-BZI complex. Green: Chain A; magenta: Chain B; red: Chain C; blue: Chain D. B. RMSDs of the TIL-L-Trp-BZI complex relative to the unliganded enzyme. The chain colors are as in A.

A

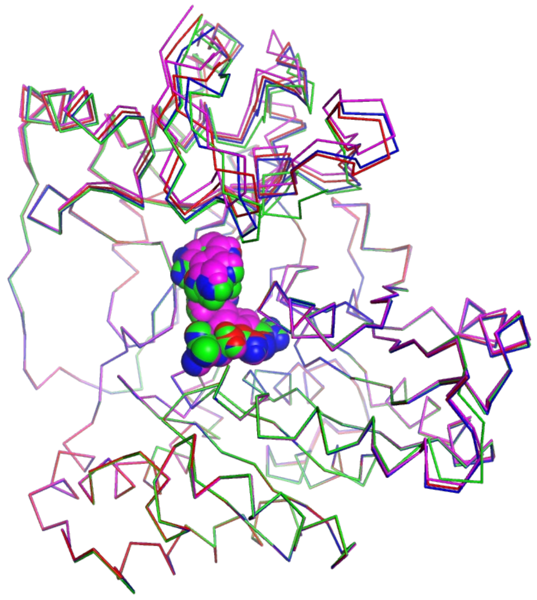

B

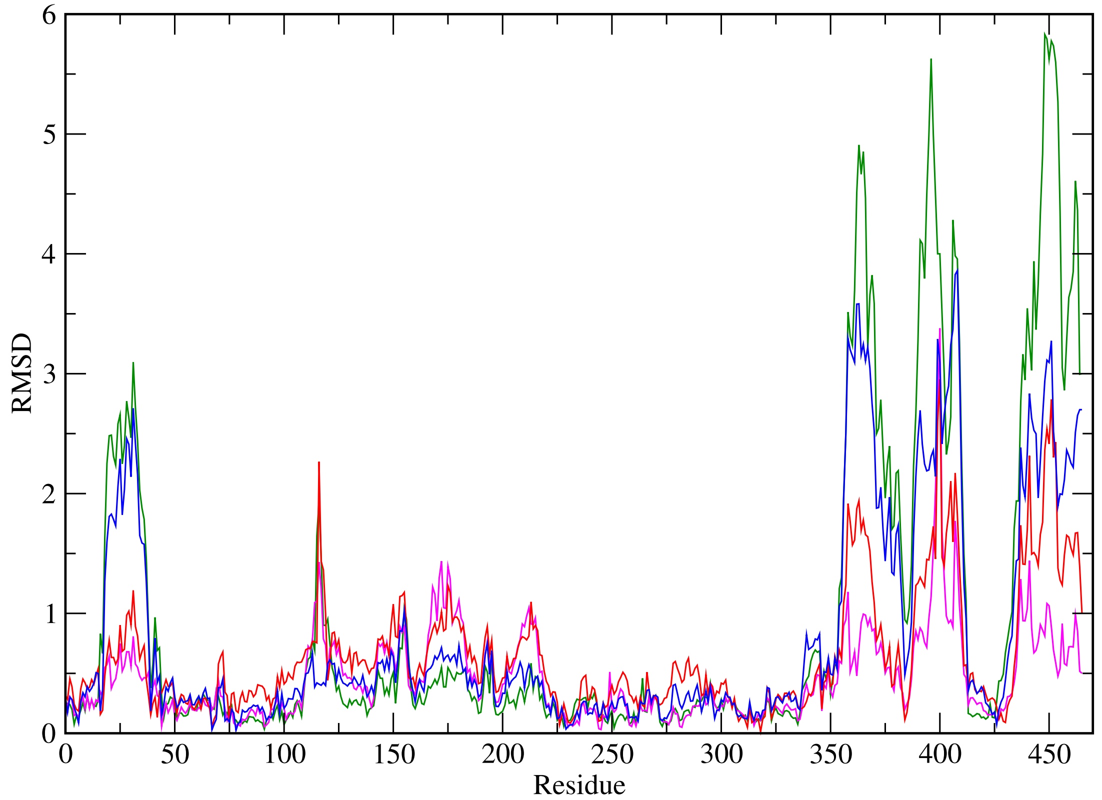

Figure S5 A. Overlay of the Cα backbones of chains of the TIL-S-Et-L-Cys-BZI complex. Green: Chain A; magenta: Chain B; red: Chain C; blue: Chain D. B. RMSDs of the TIL-S-Et-L-ys-BZI complex relative to the unliganded enzyme. The chain colors are as in A.

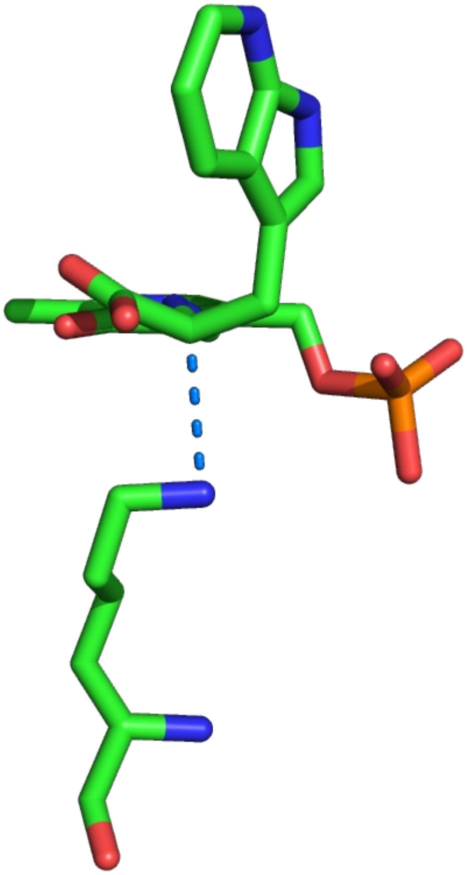

Figure S6. Structure of the external aldimine of TIL with 7-aza-L-Trp showing the allowed conformation of Lys-266 which positions it in line with the C-H bond and 3 Å away from the Cα.
